# *Achyranthes aspera* Extracts as Adjuvants for the Redressal of Antibiotic Resistance

**DOI:** 10.3390/pharmaceutics14102219

**Published:** 2022-10-18

**Authors:** Hamna Ahmad, Umar Farooq Gohar, Hamid Mukhtar, Muhammad Zia-UI-Haq, Romina Alina Marc, Marius Irimie, Luigi Geo Marceanu, Claudia Mihaela Gavris

**Affiliations:** 1 Institute of Industrial Biotechnology, Government College University Lahore, Lahore 54000, Pakistan; 2 Office of Research, Innovation and Commercialization, Lahore College for Women University, Lahore 54000, Pakistan; 3 Food Engineering Department, Faculty of Food Science and Technology, University of Agricultural Sciences and Veterinary Medicine, 400372 Cluj-Napoca, Romania; 4 Faculty of Medicine, Transilvania University of Brasov, 500036 Brasov, Romania

**Keywords:** redressal, antibiotic resistance, resistance breakers, antibiotic adjuvants, secondary metabolites, zone of inhibition

## Abstract

*Achyranthes aspera* seeds and leaves are believed to reverse antibiotic resistance and increase the efficacy of current drugs. *Achyranthes aspera* seeds and leaves contain many secondary metabolites needed for the redressal of antibiotic resistance. In the present study, seven different antibiotics were used against five different strains of bacteria such as Methicillin-resistant *Staphylococcus aureus, Enterococcus faecalis, Acinetobacter baumannii, Klebsiella pneumoniae,* and *Pseudomonas aeruginosa.* For Methicillin-resistant *Staphylococcus aureus* Cefoxitin, Penicillin, and Co-trimoxazole were resistant out of seven antibiotics. The zone of inhibition for all these three antibiotics goes from the resistant to the sensitive range after the combination with plant extracts. For *Enterococcus faecalis*, Ciprofloxacin, Levofloxacin, Penicillin, Amoxicillin, Imipenem, and Vancomycin were resistant after treatment with the plant extracts, and the Ciprofloxacin, Levofloxacin, Imipenem, and Vancomycin zones of inhibition were from the resistant to the sensitive range. An increase in zone sizes was observed for Penicillin, but it remained resistant while no zone of inhibition was observed for Amoxicillin. For *Acinetobacter baumannii*, Ciprofloxacin, Levofloxacin, Ceftriaxone, Ceftazidime, and Imipenem were resistant. After a combination of these antibiotics with plant extracts, a change in zone sizes was observed for Levofloxacin and Ceftriaxone, but it was not considerable as it remained in the resistance and intermediate ranges. No zones of inhibition were observed for Ciprofloxacin, Ceftazidime, or Imipenem. For *Klebsiella pneumoniae,* all the antibiotics were resistant. An increase in zone sizes was observed after a combination with plant extracts for Ceftazidime and Imipenem in *Klebsiella pneumoniae,* but it remained in the resistance category. No zone of inhibition was observed for *Pseudomonas aeruginosa* before or after using plant extracts against any antibiotic. This study suggests that the *Achyranthes aspera* seed and leaf extracts can reverse antibiotic resistance without any side effects on the human body, and that they can reverse antibiotic resistance naturally.

## 1. Introduction

The proliferation of pathogens by their invasion through blood circulation systems leads to severe systematic infections known as bacterial infections [1,2]. One of the biggest breakthroughs of the 20th century in medicine was the introduction of antibiotics [3]. Bacterial resistance to antibiotics is increasing with the passage of time which leads to an increase in healthcare costs and excessive morbidity and mortality rates [4]. The overuse and misuse of these treasure compounds lead to the development of AMR (antimicrobial resistance) which results in the rise of many untreatable infections [5,6,7,8,9,10]. The increase in antibiotic resistance is a serious threat to public health, either due to the hospital or community-acquired contagions of VRE (Vancomycin-Resistant *Enterococci*), VISA (Vancomycin-Intermediate *Staphylococcus aureus*), ESBL (Extended-Spectrum β-lactamases) enzyme-producing bacteria, and MRSA (Methicillin- resistant *Staphylococcus aureus*) [11]. These bacteria include *Streptococcus pneumoniae, Pseudomonas aeruginosa, Mycobacterium tuberculosis,* and *S. aureus* [12,13,14,15,16].

Compounds that potentiate the antibiotic activity when co-administered with an antibiotic are known as resistance breakers, chemosensitizers, antibiotic adjuvants, and antibiotic potentiators [17,18,19,20,21,22]. These compounds developed as a product of metabolic processes, either intermediates or as end-products. These compounds are called secondary metabolites and are biologically dynamic compounds [23,24,25,26,27,28,29]. Some of the important secondary metabolites are alkaloids, flavonoids, phenolics, tannins, polyphenols, terpenes, coumarins, quinones, lectins, saponins, etc. [30].

Antibiotic resistance reversal is very important, so that old antibiotics can be reused and the efficiency of current antibiotics can be increased. There are many synthetic, semisynthetic, and natural agents, which help in antibiotic resistance reversal [31]. Plasmid curing is the process used for the removal of plasmids from the bacterial cell. This is a much-needed process for minimizing antibiotic resistance [32]. Secondary compounds which are derived from plants show activity against resistance, such quinones (consisting of bioactive compounds major class) having the ability to eliminate plasmid [33]. *Piper nigrum, Zingiber officinale, Cinnamomum verum, Nigella sativa,* and *Plumbago zeylanica* extracts contain phenols (eugenol), saponins, naphthoquinones, flavonoids, tannins, and alkaloids which can be used as a plasmid curing agent [34].

The present study was designed to rationale the effect of the secondary metabolites present in *Achyranthes aspera* seeds and leaves as resistance breakers/adjuvants. *Achyranthes aspera,* which is commonly known as the prickly chaff flower, is a medicinal plant. Different parts of this plant’s seeds, roots, leaves, and stems are used separately for different diseases as antimicrobial, anti-cancerous, and anti-ulcer agents [35]. *Achyranthes aspera* leaves and seeds are used for the redressal of antibiotic resistance caused by multidrug-resistant bacteria.

## 2. Materials and Methods

### 2.1. Plant Material Collection

*Achyranthes aspera* leaves and seeds parts were used and collected from a local market in Lahore. The plants were identified and authenticated by the Institute of Botany Punjab University Lahore. The voucher specimen number was deposited in Bot-2256.

### 2.2. Preparation of Plant Extracts

#### 2.2.1. Cleaning and Grinding of Plant Material

The plant material was washed and dried up at 40 °C in a hot air oven. Plant material was grounded in a grinder separately. The powder obtained was passed through a sieve (mesh number @ 1.17 mm).

#### 2.2.2. Crude Extract Preparation

Methanol was chosen as the most preferable universal solvent for crude extract preparation [36]. The 200 g powdered plant material was soaked in methanol (1 L) in a proportion of 1:5, separately. Using the ultrasonic liquid processor, the methanol solutions were sonicated for 3 h, and the resulting solution was incubated in a shaker at 37 °C for 1 week and filtered (Whatman filter No. 1). The filtrate of each extract was stored for further use while the residue obtained on the filter paper was discarded. The methanol was evaporated from the filtrate by using a water bath. The evaporation of crude extracts on a water bath at 40 °C led to the formation of a semi-solid mass. After concentration, 20 g of *Achyranthes aspera* seeds and 23 g of leaves were obtained.

#### 2.2.3. Fractionation of Crude Extract

**Figure 1:**
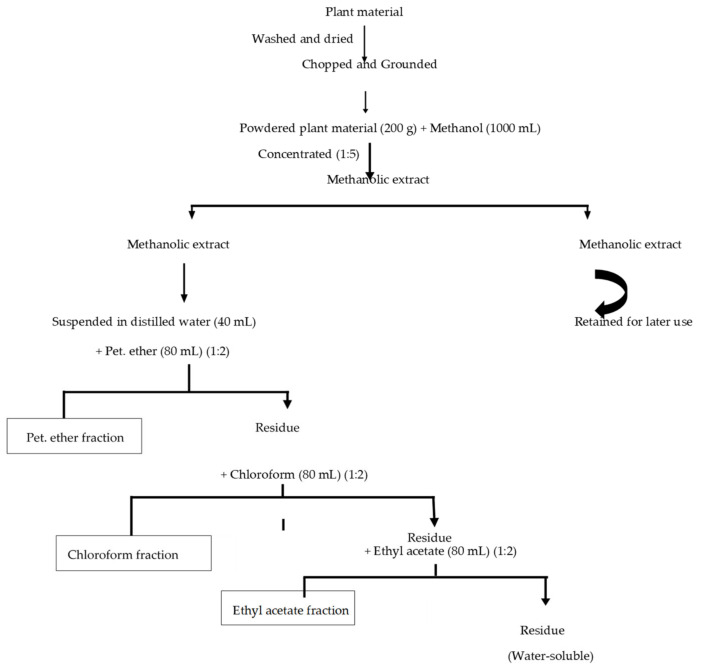
Kupchan modified protocol of extraction and fractionation protocol of *Achyranthes aspera* (seeds and leaves) [37].

Kupchan modified method [37] was used for solvent–solvent extraction. The crude methanolic extracts were fractionated into petroleum ether, chloroform, ethyl acetate, and aqueous, as shown in Figure 1.

Next, 15 g of the methanolic extract of *Achyranthes aspera* seeds was taken and dissolved in 40 mL of distilled water. This mixture was put into a separating funnel and 80 mL of petroleum ether was added in a proportion of 1:2. The mixture was shaken vigorously for almost 20 min, then the mixture was allowed to stand up to the formation of two separate layers. The layer formation is on the basis of density. From the mixture, the petroleum ether layer was separated, and is referred to as the petroleum ether fraction. It was then subjected to evaporation and after its concentration, the fraction was stored at 4 °C. The residue was saved to use in the next step.

Next, the residue was mixed with chloroform (80 mL) in a proportion of 1:2. This mixture was shaken for almost 20 min and then put to stand up to the formation of two layers. From the mixture, the chloroform layer was separated and referred to as the chloroform fraction. It was then subjected to evaporation and after its concentration, the fraction was stored at 4 °C. The residue was saved to use in the next step.

In the next step, the residue was mixed with 80 mL of ethyl acetate in a proportion of 1:2. This mixture was shaken for almost 20 min and then put to stand up to the formation of two layers. From the mixture, the ethyl acetate layer was separated and known as the ethyl acetate fraction. It was then subjected to evaporation and after its concentration, the fraction was stored at 4 °C. The residue which was water soluble was referred to as the aqueous fraction. It was then subjected to evaporation and after its concentration, the fraction was stored at 4 °C. From one part of *Achyranthes aspera,* such as its seeds, five extracts were prepared.

Next, 18 g of methanolic extract of *Achyranthes aspera* leaves was taken and dissolved in 40 mL of distilled water. This mixture was put into a separating funnel and 80 mL of petroleum ether was added in a proportion of 1:2. The same above procedure was repeated with the methanolic extract of *Achyranthes aspera* leaves and five extracts were obtained.

#### 2.2.4. Stock Preparation

All the sequential extracts obtained were dissolved in a measured quantity of DMSO (dimethyl sulfoxide) for stock preparation according to the method adopted by [38], as DMSO is a universal solvent. The DMSO was added in different volumes for different extracts according to their weight and consistency. Working solutions of different concentrations were prepared from these stock solutions using DMSO. These working solutions were stored in glass vials at 4 °C for further studies.

### 2.3. Phytochemical Screening

Phytochemical screening was performed for the above-mentioned extracts of *Achyranthes aspera* seeds and leaves for the confirmation of secondary metabolites by using standard procedures [39]. For the confirmation of alkaloids, firstly, Wagner’s test was performed with all plant extracts using the method adopted by [40]. For further confirmation, a Mayer’s test and Tannic acid test were performed using the method of [41]. For the confirmation of variable carbohydrates, Barfoed’s test, Fehling’s test, and Molish tests were performed using the methods given by [42]. A cardiac test was performed for glycoside confirmation by adopting the same method as shared by [37]. A Legal’s test was also performed for the confirmation of glycosides by using the method of [43]. For the confirmation of proteins, two types of tests were performed, i.e., the Biuret test and Ninhydrin test, using the methods given by [42] and [44], respectively.

A spot test was performed for the confirmation of fixed oils and fats by adopting the procedure given by [45]. For tannins, a gelatin test was performed by using the method documented by [46]. For the confirmation of terpenes, the test method used was given by [39]. For flavonoid confirmation, the test method used was given by [47]. Foam test was performed for the confirmation of saponins the method adopted was developed by [41]. For steroids, a Libermann–Burchard reaction test was performed by adopting the procedure of [42]. For anthraquinone and coumarin confirmation, the method used was the same as that adopted by [48]. For the confirmation of phlobatannins, emodins, and anthocyanin, tests were performed by using the method given by [49].

### 2.4. Bacterial Strains Collection

Five bacterial strains, *Staphylococcus aureus, Acinetobacter baumannii, Enterococcus faecalis, Klebsiella pneumoniae,* and *Pseudomonas aeruginosa,* were collected from the pathology laboratory of Nawaz Sharif Social Security Hospital Lahore. Gram staining was performed for confirming the bacterial strains. For culture characteristics, strains were streaked on different media and biochemical tests were performed. All confirmation processes were performed at the Nawaz Sharif Social Security Hospital of Lahore.

### 2.5. Confirmation of Resistant Bacterial Strains

The disc diffusion method was used for the AST test to confirm the resistance of respective bacterial strains [50]. Mueller–Hinton agar (MHA) plates were prepared and antibacterial susceptibility testing (AST) was performed. Bacterial suspension in sterilized normal saline matched to the McFarland standard (0.5) was prepared and OD600 was noted. For a lawn of growth on MHA plates, 10–15 µL into bacterial suspension was poured and streaked onto the Mueller–Hinton agar plate. After streaking plates, antibiotic discs were placed on the bacterial lawn with forceps and were gently pressed. Then plates were incubated at 37 °C for 24 h. After incubation, the zone of inhibition was measured by using a metric ruler. Then, the zone of inhibition was compared with a standard zone of inhibition given by the Clinical Laboratory Standard Institute 2020 [51].

### 2.6. Antibiotic Susceptibility Test for Plant Extracts

The disc diffusion method was used for the AST test to confirm the resistance of respective bacterial strains to plant extracts [50]. Mueller–Hinton agar (MHA) plates were prepared, and antibacterial susceptibility testing (AST) was performed. Bacterial suspension in sterilized normal saline matched to the McFarland standard (0.5) was prepared and OD600 was noted. For a lawn of growth on MHA plates, 10–15 µL bacterial suspension was poured and streaked onto the Mueller–Hinton agar plate. Then, the plate was dried for 5 min. After this step, filter paper discs containing 10–15 µL of the respective extract were placed on the surface of the agar media with the help of simple forceps and were gently pressed. Then the plates were incubated at 37 °C for 24 h. Then after incubation, a metric ruler was used for the measurement of zone of inhibition of each antibiotic.

### 2.7. Antibiotic Resistance Redressal Activity

Plant extracts in combination with antibiotics were checked for the antibiotic resistance redressal activity. Then, 500 mL Mueller–Hinton (MHA) agar was prepared in a flask and autoclaved, after which 12–15 mL MHA agar was poured in Petri plates. Next, 200 mL nutrient broth was prepared and autoclaved, after which 10 mL nutrient broth was poured in all test tubes. Five test tubes were inoculated with each of the five bacterial strains such as Methicillin-resistant *Staphylococcus aureus, Enterococcus faecalis, Acinetobacter baumannii, Klebsiella pneumoniae*, and *Pseudomonas aeruginosa* to form bacterial suspension compared with the bacterial suspension with McFarland (0.5) and OD600. Five extracts were prepared from each part of the plant and all these extracts were used for testing redressal activity. For the treatment of bacterial culture with plant extract in a test tube, 10 mL of nutrient broth and almost 200 µL of bacterial suspension were added so that the OD600 could be compared with the control. Then, 1 mL of each plant extract was added to the respective test tube, and the final OD600 was noted. Test tubes containing extracts and bacterial suspension were cotton-plugged and put in a static incubator overnight at 37 °C. After incubation, the OD600 was again noted and compared to the OD600 before incubation. Mostly, the OD600 remained the same as the bacteria, showing very little or no growth when put in the static incubator with plant extracts. For mixtures that showed an increase in OD600, some nutrient broth was added so that the OD600 would be the same as before incubation. Then, 10–15 µL bacterial and extract suspensions from each test tube were poured onto the MHA plates with a micropipette. The suspension was spread onto the Mueller–Hinton agar plate by using sterilized cotton swabs. The plates were dried for 2 min and antibiotic discs were placed on agar plates with the help of sterilized forceps, and the discs were pressed gently. These plates were then incubated in an incubator at 37 °C for 24 h. After incubation, the zones of inhibition of each antibiotic were measured with the help of a metric ruler, and the difference in the zones of inhibition from the AST test results of plant extracts and antibiotics alone were both used as the control.

## 3. Results and Discussion

### 3.1. Phytochemical Screening

#### 3.1.1. For *Achyranthes aspera* (Seed)

Table 1 shows the results of the phytochemical screening of *Achyranthes aspera* seeds. The test results of these extracts revealed the presence of many bioactive compounds that could be important for their numerous medicinal properties [49].

The results showed the presence of almost all important bioactive compounds which were being tested. These compounds were observed in variable concentrations in different seed extracts of *Achyranthes aspera*. Emodins, anthocyanins, and anthraquinones were never observed in any seed extract of *A. aspera*. Most of the bioactive compounds were observed in the methanolic and ethanolic extracts of the respective parts of the plant.

#### 3.1.2. For *Achyranthes aspera* Leaves

Table 2 shows the results of the phytochemical screening of *Achyranthes aspera* leaves. The test results of these extracts revealed the presence of many bioactive compounds that could be important for their numerous medicinal properties [49].

The results showed the presence of almost all important bioactive compounds which were being tested.

The qualitative studies of the above–mentioned extracts show the presence of many bioactive compounds, such as tannins which play a role in antitumor, antimicrobial, and antiviral activity [52,53]. They have also been reported as an inhibitory agent in HIV replication [54]. They are important for the healing of mucous membranes [55]. The presence of cardiac glycosides indicated their use in many heart diseases [56]. The presence of phenols was indicated by many plant extracts. They act as antioxidants in humans as well as plants [57]. Saponins act as an important tool in the defense mechanism of plants as they inhibit microbial attacks, and they can be used for the treatment of fungal as well as yeast infections [58]. Alkaloids play an important role in many pharmacological actions. They can act as antimicrobial, antimalarial, anti-hyperglycemic, and anti-cancerous agents [59]. They can be exploited for recreational drugs [60]. Terpenes could act as a therapeutic as well as a protective agent. They could be used in the agriculture industry for storing agricultural products [61]. Steroids play their role in the proper regulation of immune responses [62].

Phlobatannins show many astringent properties [63]. Carbohydrates provide strength for the different functions of the body; thus, we can say that they act as petrol to strengthen the body [64]. Due to the antimicrobial and anti-inflammatory actions of coumarins, they are thought to have anti-hyperproliferative effects in skin diseases [65]. Proteins’ nutritional value could not be declined, as they act as central units or building blocks for life. For the maintenance of life and for a healthier body, proteins are a much-needed component [66].

Previous research has also suggested the presence of tannins in *Achyranthes aspera* seed and leaf extracts [67], and the presence of phenols and saponins in *Achyranthes aspera* seed extracts [68]. In [69], the authors also reported the presence of glycosides in *Achyranthes aspera* seed extracts, and [12] also reported the presence of glycosides, terpenes, carbohydrates, proteins, and phenols in *Achyranthes aspera* leaf extracts. In [56], the authors also suggested the presence of alkaloids, steroids, and saponins in *Achyranthes aspera* leavf extracts, and [70] also suggested the presence of alkaloids, carbohydrates, and coumarins in *Achyranthes aspera* seed extracts. In [60], the authors also reported the presence of terpenes, proteins, and steroids in *Achyranthes aspera* seed extracts, and [60] also reported the presence of phlobatannins in *Achyranthes aspera* seeds and leaf extracts. In [71], the authors suggested that most of the bioactive compounds could be perceive as a natural source of antibiotics and could provide assistance to the body from microbial invasion as well as bacterial attack. From the current exploration, the medicinal properties of the above-mentioned plant could be explored on the basis of the presence of different chemical constituents in different plant extracts. These bioactive constituents could be an important source for the redressal of antibiotic resistance and could act as resistance breakers.

### 3.2. Confirmation of Resistant Bacterial Strains

Antibiotic susceptibility tests (AST) were used for the confirmation of resistant bacterial strains. Respective bacterial colonies were streaked on agar plates by using sterile swabs, and after putting antibiotic discs on the bacterial-streaked plates, they were incubated at 37 °C for 24 h. After incubation, the zone sizes were measured for each antibiotic against the respective bacterial strains.

#### 3.2.1. AST Test of *Staphylococcus aureus*

Table 3 shows the results for the *Staphylococcus aureus* antibiotic susceptibility test, in which the zone of inhibition for Ciprofloxacin was 21 mm. For Levofloxacin, it was 14 mm, for Amikacin and Cefoxitin it was 17 mm, and for Linezolid it was 24 mm. No zone of inhibition was shown by Penicillin or Co-trimoxazole. From Table 3, it can be observed that the zone sizes for the resistant-category Linezolid, Ciprofloxacin, Levofloxacin, Penicillin, Amikacin, Cefoxitin, and Co-trimoxazole should be less than or equal to 20, 15, 12, 12, 13, 21, and 10 mm, respectively [72]. However, the zone sizes for the tested organism lay in the intermediate as well as the sensitive range.

#### 3.2.2. AST Test of *Enterococcus faecalis*

Table 4 shows the results for the *Enterococcus faecalis* antibiotic susceptibility results, in which the zone of inhibition for Ciprofloxacin was 13 mm. For Levofloxacin, it was 10 mm, for Imipenem and Vancomycin it was 14 and 10 mm, respectively, and for Linezolid it was 19 mm. No zone of inhibition was shown by Amoxicillin. From Table 4, it can be observed that the zone sizes for Linezolid, Ciprofloxacin, Levofloxacin, Penicillin, Amoxicillin, Imipenem, and Vancomycin should be less than or equal to 20, 15, 13, 12, 13, 18, and 14 mm, respectively [72]. The *Enterococcus faecalis* tested strain showed zones that were resistant to all respective antibiotics, as it was considered an MDR (multidrug-resistant bacteria). It was also a Vancomycin-resistant *Enterococcus faecalis* strain.

#### 3.2.3. AST of *Acinetobacter baumannii*

Table 5 shows the results for the *Acinetobacter baumannii* antibiotic susceptibility results, in which the zone of inhibition for Levofloxacin was 9 mm, and for Amikacin and Co-trimoxazole it was 18 and 19 mm, respectively. No zone of inhibition was shown by Ciprofloxacin, Ceftriaxone, Ceftazidime, or Imipenem for *Acinetobacter baumannii.*

From Table 5, it can be observed that the zone sizes for Ciprofloxacin, Ceftriaxone, Levofloxacin, Ceftazidime, Amikacin, Imipenem, and Vancomycin should be less than or equal to 21, 19, 16, 17, 13, 19 and 10 mm, respectively [72]. The *Acinetobacter baumannii* tested strain showed zones that were resistant to all respective antibiotics as it was considered an MDR (multidrug-resistant bacteria).

#### 3.2.4. AST of *Klebsiella pneumoniae*

Table 6 shows the results for *Klebsiella pneumoniae.* No zone of inhibition was shown by any of the following antibiotics: Levofloxacin, Amikacin, Co-trimoxazole, Ciprofloxacin, Ceftriaxone, Ceftazidime, or Imipenem. From Table 6, it can be observed that the zone sizes for Ciprofloxacin, Ceftriaxone, Levofloxacin, Ceftazidime, Amikacin, Imipenem, and Vancomycin should be less than or equal to 21, 19, 16, 17, 13, 19, and 10 mm, respectively [72]. The *Klebsiella pneumoniae* tested strain showed zones that were resistant to all respective antibiotics as it was considered an MDR (multidrug-resistant bacteria).

#### 3.2.5. AST of *Pseudomonas aeruginosa*

Table 7 shows the results for the *Pseudomonas aeruginosa* antibiotic susceptibility test results, wherein the zones of inhibition for Levofloxacin, Amikacin, Co-trimoxazole, Ciprofloxacin, Ceftriaxone, Ceftazidime, and Imipenem were examined. No zone of inhibition was shown by any of the tested antibiotics.

From Table 7, it can be observed that the zone sizes for Ciprofloxacin, Ceftriaxone, Levofloxacin, Ceftazidime, Amikacin, Imipenem, and Vancomycin should be less than or equal to 21, 19, 16, 17, 13, 19, and 10 mm, respectively [72]. The *Pseudomonas aeruginosa* tested strain showed zones that were resistant to all respective antibiotics as it was considered an MDR (multidrug-resistant bacteria).

### 3.3. AST (Antibiotic Susceptibility Test) for Plant Extracts

Antibiotic susceptibility tests (AST) were used for the confirmation of resistant bacterial strains. Respective bacterial colonies were streaked on agar plates by using sterile swabs, and after putting discs soaked in plant extracts on the bacterial streaked plates, they were incubated at 37 °C for 24 h. After incubation, the zone sizes were measured for each antibiotic against the respective bacterial strains.

#### 3.3.1. AST Results for *Achyranthes aspera* Seed

Antibiotic susceptibility test results in Table 8 showed zone of inhibition for *Achyranthes aspera* seed extracts. No zone of inhibition was shown by any of the following extracts—methanolic, petroleum ether, chloroform, ethyl acetate, or aqueous—against the tested bacteria. Thus, it was concluded that plant extracts had no antimicrobial potential for the multidrug-resistant bacterial strains.

#### 3.3.2. AST Results for *Achyranthes aspera* Leaves

Table 9 shows the results for the *Achyranthes aspera* leaves. No zone of inhibition was shown by any of the following extracts—methanolic, petroleum ether, chloroform, ethyl acetate, and aqueous—against tested bacteria. Thus, it was concluded that plant extracts had no antimicrobial potential for the multidrug-resistant bacterial strains.

The composition of the sequential extract deals with the activity of these extracts [73]. The antimicrobial activity of the sequential extracts also depends upon the water and alcoholic content of the extract. The biological activities of the active compounds present in an extract showed different activities depending upon the chemical composition and environment of the extracts in which they are prepared [74]. The plant extracts can be used synergistically to enhance the effect of antibiotics used against specific bacteria. They may not kill bacteria by themselves, but they can potentiate the antibiotic effect. With the emergence of antibiotic resistance, the need for screening medicinal plants enhances [75]. Thus, the plants in the present study did not show antimicrobial activity, but they could be used as resistant breakers or resistant adjuvants for the redressal of resistance against different classes of antibiotics.

### 3.4. Redressal Activity of Antibiotic Resistance for MRSA

#### 3.4.1. Ciprofloxacin

**Figure 2:**
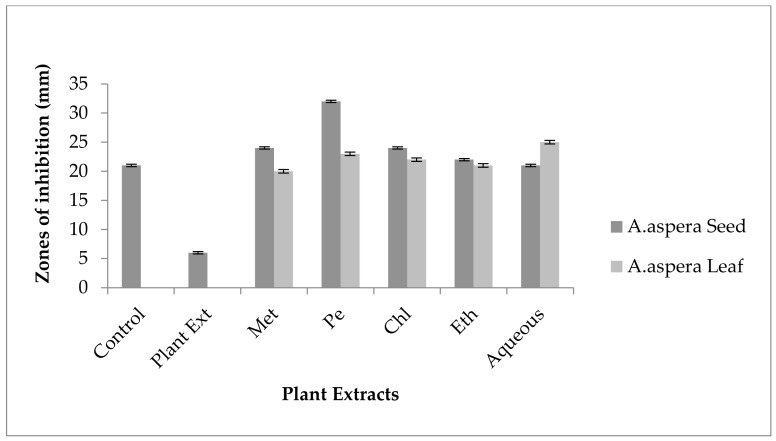
Redressal of Ciprofloxacin resistance in *MRSA* by using Ciprofloxacin in combination with various plant extracts of *Achyranthes aspera* (leaves and seeds). All the values are means of three parallel replicates. Error in mean values indicated by error bars.

Ciprofloxacin showed a 21 mm inhibition zone without treating it with plant extracts for *MRSA*. From the standard, it was concluded that the zone size should be above or equal to 21 mm to be called a sensitive zone. Thus, Ciprofloxacin was sensitive to *MRSA*. No zone of inhibition was shown by any plant extract alone. After the combination of antibiotics with plant extracts, an increase in the zone size was observed. The zone of inhibition was measured at 32 mm with *Achyranthes aspera* petroleum ether seed extract, which was the highest zone for *Achyranthes aspera* seeds. The zones of inhibition for *Achyranthes aspera* seed methanolic and chloroform extracts increased up to 24 mm, while for *Achyranthes aspera* seed ethyl acetate extract it was 22 mm and for water extract no change in zone size was observed, as shown in Figure 2. The zone of inhibition was measured as 25 mm with *Achyranthes aspera* leaf aqueous extract, which was the highest zone for *Achyranthes aspera* leaves. The zones of inhibition for *Achyranthes aspera* leaf methanolic, chloroform, and petroleum ether extracts increased up to 20, 22, and 20 mm, respectively, while for *Achyranthes aspera* leaf ethyl acetate extract it was 21, and no change in zone size was observed.

Moveable genetic elements, which lead to the horizontal transfer of resistant genes [76], and efflux pump upregulation are the two main reasons for resistance in *MRSA* [77]. This antibiotic belongs to the class of antibiotics known as Fluoroquinolones. They work by inhibiting the synthesis of DNA in bacterial cells, to make bonds with DNA gyrase and enzymes topoisomerases IV [78]. The resistance to Ciprofloxacin in bacteria is acquired mainly by two mechanisms: One is through the modification of the target site, and the other is by the activation of efflux pumps. Target site modification is done by the modification of *gyrAB* as well as *parCE*, by which the affinity of Ciprofloxacin decreases for DNA gyrase and enzyme topoisomerase IV. The activation of efflux pumps inhibits the entry of antibiotics into the bacterial cell [79]. After treatment with plant extracts, an increase in zone size can be seen, which means that the plant extracts potentiate the activity of Ciprofloxacin.

#### 3.4.2. Amikacin

**Figure 3:**
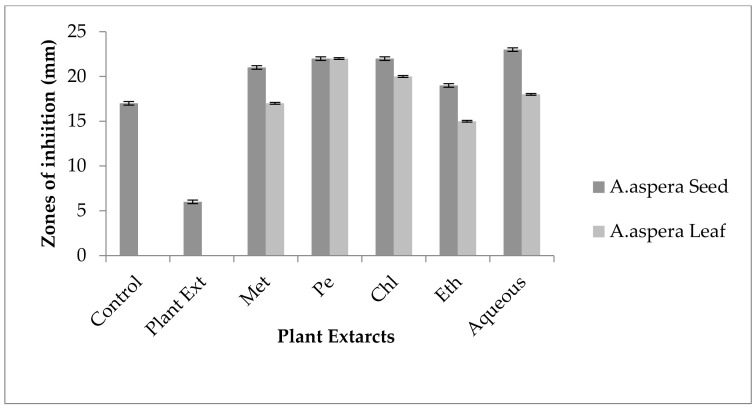
Redressal of Amikacin resistance in *MRSA* by using Amikacin in combination with various plant extracts of *Achyranthes aspera* (leaves and seeds).

No inhibition zone was shown by any plant extract alone for *MRSA*, while the inhibition zone with Amikacin was 17 mm without plant extract treatment. When compared with the standard, the inhibition zone must be equivalent to or overhead 18 mm to be measured as a sensitive zone. Amikacin showed an intermediate zone of inhibition. After combination with plant extracts, the AST results showed redressal in resistance towards Amikacin. The inhibition zone was measured at 23 mm with *Achyranthes aspera* aqueous seed extract, which was the highest zone for *Achyranthes aspera* seeds. The zones of inhibition for *Achyranthes aspera* seed methanolic, chloroform, petroleum ether, and ethyl acetate extracts increased up to 21, 22, 22, and 19 mm, respectively. In Amikacin, the zone of inhibition went from intermediate resistant to the sensitive range. The highest zone of inhibition was measured at 24 mm with *Achyranthes aspera* leaves for petroleum ether extract. The zones of inhibition for *Achyranthes aspera* leaf methanolic chloroform, ethyl acetate, and aqueous extracts were 15, 23, 18, and 22 mm, respectively, which can be seen in Figure 3.

It is semisynthetic antibiotics which belong to the aminoglycoside class of antibiotics. Amikacin binds to 16S RNA on the A site which leads to the inhibition of protein synthesis. *ACC(6′)-Ie* is the type of enzyme which is involved with the acetylation process for aminoglycoside which is considered an important resistance mechanism for Amikacin. This enzyme could present both in chromosome and plasmids. The inhibition of the gene that encodes for the above-mentioned enzyme leads to the reversal of Amikacin resistance in many organisms [80]. From the results of present study, it was concluded that the Amikacin zones of inhibition went from the resistant to the sensitive range. The resistance of Amikacin might be reversed due to the inhibition of enzymes encoding genes.

#### 3.4.3. Cefoxitin

**Figure 4:**
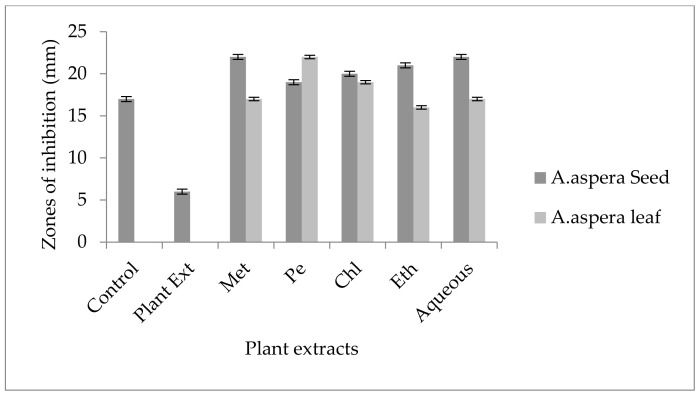
Redressal of Cefoxitin resistance in *MRSA* by using Cefoxitin in combination with various plant extracts of *Achyranthes aspera* (leaves and seeds).

Cefoxitin showed a 17 mm inhibition zone without treating it with plant extracts for *MRSA*. From the standard, it was concluded that the zone size should be above or equal to 22 mm to be called a sensitive zone. No zone of inhibition was shown by any plant extract alone. After the combination of antibiotics with plant extracts, an increase in zone sizes was observed by some plant extracts. The zones of inhibition for *Achyranthes aspera* seed methanolic, petroleum ether, chloroform, ethyl acetate, and aqueous extracts were 22, 19, 20, 21, and22 mm, respectively. No zone of inhibition was shown by any plant extract alone. For *Achyranthes aspera* leaves, the highest zone was observed by petroleum ether extract. Then for chloroform extract, which was 19 mm, for aqueous and methanolic and ethyl acetate extracts, no change in zone size was observed, as shown in Figure 4.

This antibiotic belongs to the β-Lactam class of antibiotics. Cefoxitin inhibits the synthesis of the cell wall. It usually binds with PBPs (Penicillin-binding proteins). They themselves are resistant to the β-Lactam ring degrading enzymes known as β-Lactamases. To resist Cefoxitin PBP2a proteins, a modified form of PBPs with no binding site for antibiotic attachment is activated by the regulation of *mecA* genes [81]. Resistance against Cefoxitin could be reversed by the suppression of genes encoding for PBP2a proteins. From the above-mentioned results, it can be concluded that the plant extracts play their role in the redressal of antibiotic resistance in *MRSA*. The bioactive compounds present in plant extracts might suppress the PBP2a-encoding genes due to which the zone of inhibition goes from the resistant to the sensitive range.

#### 3.4.4. Levofloxacin

**Figure 5:**
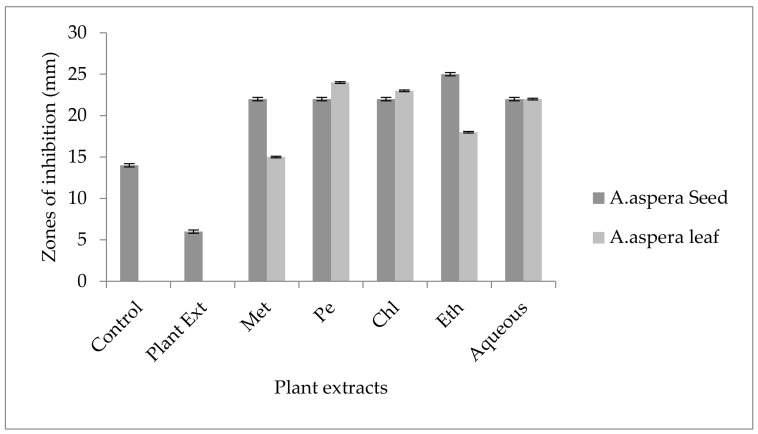
Redressal of Levofloxacin resistance in *MRSA* by using Levofloxacin in combination with various plant extracts of *Achyranthes aspera* (leaves and seeds).

In *MRSA*, the inhibition zone of Levofloxacin was 14 mm before treatment with plant extracts. According to the standard, the inhibition zone must be overhead or equivalent to 15 mm to be reflected as a sensitive zone. Thus, Levofloxacin was of intermediate resistance to *MRSA*. No change in zone size was observed with methanolic, petroleum ether, chloroform, and aqueous extracts of any plant part alone. The AST results showed a considerable increase in zone size after the antibiotic and plant extract combination. The zone of inhibition was measured as 25 mm with *Achyranthes aspera* ethyl acetate seed extract, which was the highest zone for *Achyranthes aspera* seeds. The zones of inhibition for *Achyranthes aspera* seed methanolic, chloroform, petroleum ether, and aqueous extracts increased up to 22 mm. With plant extracts of seeds, the zone for Levofloxacin went from intermediate to sensitive, as shown in Figure 5. The zone of inhibition was measured as 24 mm with *Achyranthes aspera* leaf petroleum ether extract, which was the highest zone for *Achyranthes aspera* leaves. The zones of inhibition for *Achyranthes aspera* leaf methanolic and chloroform extracts increased up to 20 and 22 mm, respectively, while for *Achyranthes aspera* leaf methanolic extract it was 15 mm.

Resistance to Levofloxacin can be reversed by inhibiting the efflux pumps, which is possible with the help of EPIs (efflux pump inhibitors). These EPIs either synergize the effect of antibiotics or play their role in the re-sensitization of bacteria for Levofloxacin [82]. The inhibition zones of Levofloxacin increased considerably for *MRSA* after the combination with plant extracts. The zone sizes went from the resistant to the sensitive range. From the results, it can be concluded that these plant extracts might have EPIs that reverse the effect of active efflux pumps.

#### 3.4.5. Penicillin

**Figure 6:**
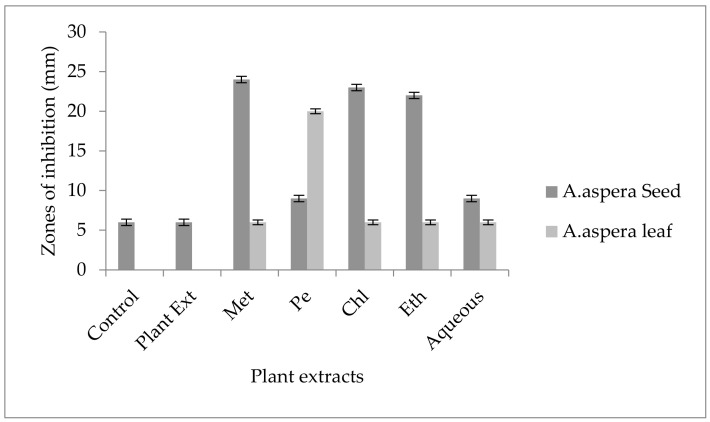
Redressal of Penicillin resistance in *MRSA* by using Penicillin in combination with various plant extracts of *Achyranthes aspera* (leaves and seeds).

Penicillin showed no inhibition zone without treatment with the plant extracts, as shown in Figure 6. In comparison with the standard given by CLSI (2020), the inhibition zone should be equal to or above 15 mm for falling in the sensitive range. After treatment with the *Achyranthes aspera* seed extracts, the zone size increased up to 24 mm with the methanolic extract, which lay within the sensitive zone range. The zone sizes increased up to 20, 23, 22, and 9 mm with petroleum ether, chloroform, ethyl acetate, and aqueous seed extracts, respectively. No zone of inhibition was shown by any plant extract alone. When the plant extracts of *Achyranthes aspera* leaves combined with Penicillin, the highest change in the zone of inhibition was observed, up to 20 mm by petroleum ether extract, while no change in zone sizes with methanolic, chloroform ethyl acetate, or aqueous extracts was observed.

Penicillin belongs to the family of antibiotics known as β-lactam. The name was given due to the presence of the β-lactam ring. It is a four-membered ring. Penicillin performs its function by inhibiting the synthesis of the cell wall. In *MRSA,* the resistance of Penicillin is due to the production of enzymes known as β-lactamase. These enzymes resist antibiotics by destroying their ring structure [83]. Penicillin was resistant to *MRSA,* and by using the above-mentioned extracts, it became sensitive to the respective bacteria. The plant extracts either synergizedthe effect of antibiotics or re-sensitized the bacteria to the respective antibiotics. From the results, it was concluded that the plant extracts reversed the resistance mechanism of *MRSA* against Penicillin.

#### 3.4.6. Linezolid

**Figure 7:**
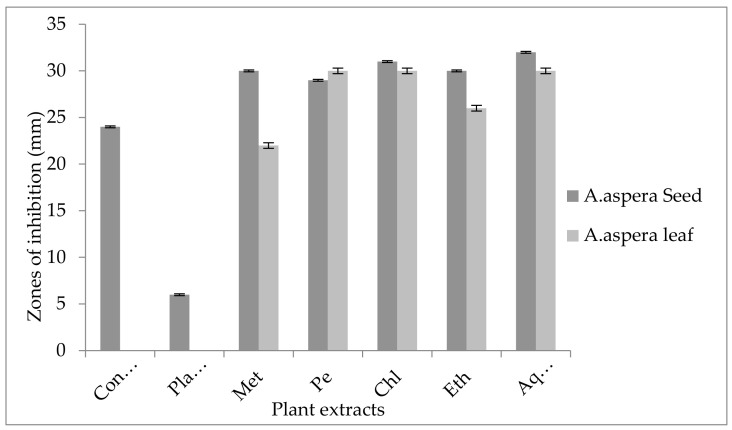
Redressal of Linezolid resistance in *MRSA* by using Linezolid in combination with various plant extracts of *Achyranthes aspera* (leaves and seeds).

The Linezolid zone of inhibition for *MRSA* was 24 mm before treatment with plant extracts. In comparison with the standard, the inhibition zone should be equal to or above 21 mm to fall into the sensitive range. After treatment with the plant extracts of *Achyranthes aspera* seeds, the zone sizes increased. The highest zone size was measured for the aqueous extract of seeds, which was 32 mm. The zones sizes for the methanolic, petroleum ether, chloroform, and ethyl acetate extracts were 30, 29, 31, and 30 mm, respectively. No zone of inhibition was shown by any plant extract alone as shown in Figure 7. The zone sizes increase for the *Achyranthes aspera* leaves methanolic, petroleum ether, chloroform, ethyl acetate, and aqueous extracts up to 22, 30, 30, 26, and 30 mm, respectively.

Linezolid belongs to the antibiotic class known as oxazolidinone. It is mainly used for the treatment of nosocomial infections which are difficult to treat mainly due to *MRSA* [84]. It is a synthetic antibiotic. Linezolid binds to the PTC, showing an overlap for the binding site of Clindamycin and Chloramphenicol. It interferes with the aa-tRNA, especially with the amino–acyl moiety, due to which it inhibits the formation of peptide bonds as well as peptidyl transferase [85,86]. Resistance against Linezolid is quite rare up to date. However, recently, some cases have reported its resistance, which has been due to the accumulation of plasmids encoding for *Cfr* resistance [87]. From the above-mentioned results, it was concluded that by using plant extracts, the effect of Linezolid was enhanced. Linezolid was sensitive to the respective strain, but plant extracts potentiate the effect of Linezolid to a great extent.

#### 3.4.7. Co-trimoxazole

**Figure 8:**
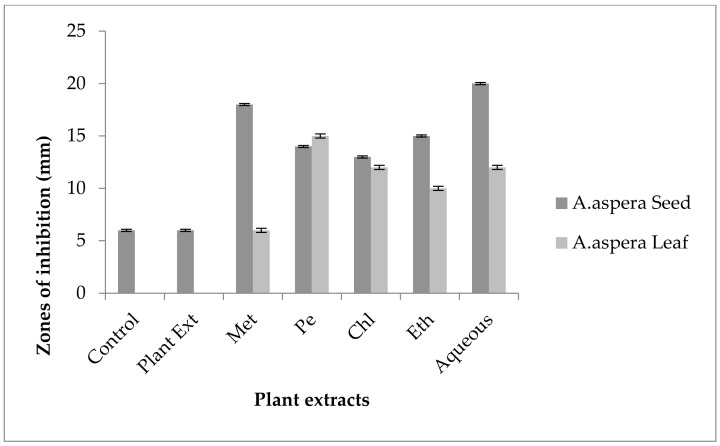
Redressal of Co-trimoxazole resistance in *MRSA* by using Co-trimoxazole in combination with various plant extracts of *Achyranthes aspera* (leaves and seeds).

For *MRSA,* no zone of inhibition was shown by any plant extract alone. No zone of inhibition of Co-trimoxazole against *MRSA* was shown. To be sensitive against the respective bacteria, the zone size should be equal to or above 16 mm, according to the standard table provided by CLSI (2020). After the combination of antibiotics with the plant extract of *Achyranthes aspera* seeds, an increase in the zone size was observed. The highest zone was observed with the aqueous extract of seeds, which is 20 mm. The zone sizes for the methanolic, petroleum ether, chloroform, and ethyl acetate extracts were 18, 14, 13, and15 mm, respectively, as shown in Figure 8. *Achyranthes aspera* leaf extracts also showed an increase in the zone sizes for petroleum ether, chloroform, ethyl acetate, and aqueous extracts up to 15, 12, 10, and 12 mm, respectively. While in combination with methanolic extracts, no change in zone size was observed, as shown in Figure 8.

Co-trimoxazole belongs to the sulfonamide group of antibiotics. They perform their function by inhibiting the synthesis of folate by directly acting as a competitor of PABA (p-aminobenzoic acid) [88]. This antibiotic is actually a combination of Trimethoprim and Sulfamethoxazole, mainly used for the curing of STIs caused by *MRSA*. *MRSA* emerges as resistant against Co-trimoxazole with the chromosomal mutations in genes encoding for *DHRF* and *DHPS* [89]. From the results, it was concluded that with the use of the strain of *MRSA* resistant to Co-trimoxazole, when the plant extracts were used, the zone size increased and it went into the sensitive zone range with many plant extracts. From the results, it was concluded that the plant extract redressal led to antibiotic resistance in *MRSA.*

### 3.5. Antibiotic Resistant Redressal Activity for Enterococcus faecalis

#### 3.5.1. Ciprofloxacin

**Figure 9:**
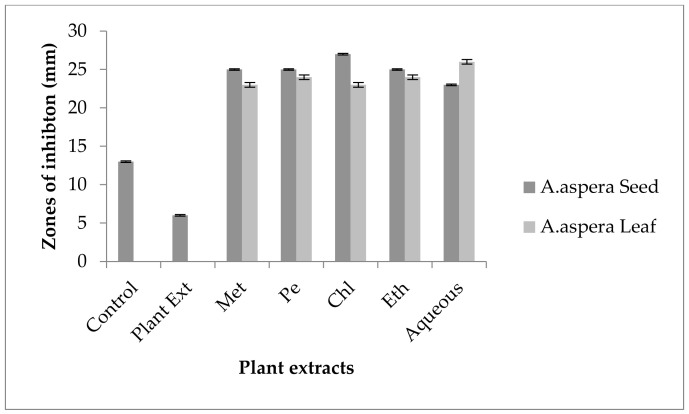
Redressal of Ciprofloxacin resistance in *Enterococcus faecalis* by using Ciprofloxacin in combination with various plant extracts of *Achyranthes aspera* (leaves and seeds).

For *Enterococcus faecalis*, Ciprofloxacin showed an inhibition zone of 13 mm without treatment with any plant extracts. In comparison with the standard given by CLSI (2020), the inhibition zone should be above or equal to 21 mm to fall into the sensitive zone range. Thus, Ciprofloxacin was sensitive to *Enterococcus faecalis*. No zone of inhibition was shown by any plant extract alone. The AST results exhibited a noticeable increase in zone size after the combination of antibiotics with plant extracts. The size of inhibition was measured as 27 mm with *Achyranthes aspera* seed chloroform extract, which was the highest zone. The zones of inhibition for *Achyranthes aspera* seed methanolic and petroleum ether and ethyl acetate extracts increased up to 25 mm, while for *Achyranthes aspera* seed aqueous extract it was 23 mm, as shown in Figure 9. The zone of inhibition was measured as 26 mm with *Achyranthes aspera* leaf aqueous extract which was the highest zone. The zones of inhibition for *Achyranthes aspera* leaf petroleum ether and ethyl acetate extracts increased up to 24 mm. For *Achyranthes aspera* leaf methanolic and chloroform extract, it was 23 mm, as shown in Figure 9.

Resistance in *Enterococcus faecalis* develops due to the presence of multidrug-resistant genes, which relate to chromosomes or plasmids [90]. Ciprofloxacin was resistant to *Enterococcus faecalis*, and by using the above-mentioned extracts, it became sensitive to the respective bacteria. The plant extracts either synergized the effect of antibiotics or re-sensitized the bacteria to the respective antibiotic. From the results, it was concluded that the plant extracts reverse the resistance mechanism of *Enterococcus faecalis* against Ciprofloxacin.

#### 3.5.2. Amoxicillin–Clavulanate

**Figure 10:**
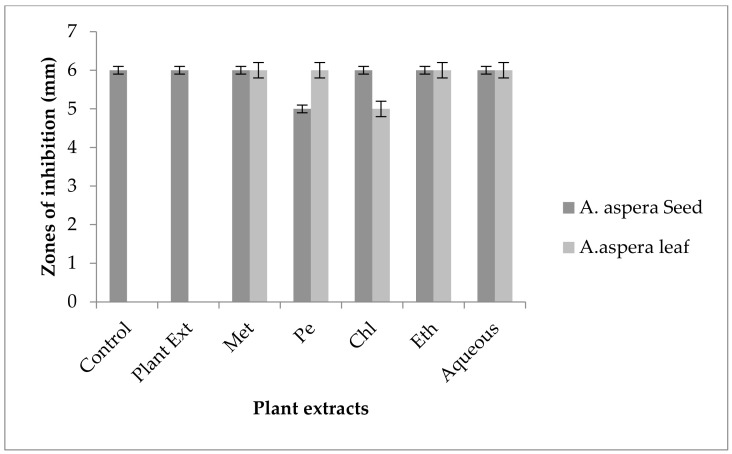
Redressal of Amoxicillin–Clavulanate resistance in *Enterococcus faecalis* by using Amoxicillin–Clavulanate in combination with various plant extracts of *Achyranthes aspera* (leaves and seeds).

Without treatment by plant extracts, no inhibition zone of Amoxicillin for *Enterococcus faecalis* was observed. In comparison with the standard given by CLSI (2020), the inhibition zone must be above overhead or equal to 18 mm to lie in the sensitive category. With treatment by plant extracts, the AST results directed by the inhibition zone with *Achyranthes aspera* seed extracts did not change at all, which meant that these extracts did not have an increasing effect with Amoxicillin, as shown in Figure 10. No zone of inhibition was shown by any plant extract alone. Methanolic, petroleum ether, chloroform, ethyl acetate, and aqueous extracts of *Achyranthes aspera* leaves showed no activity either alone or in combination with the antibiotic, as shown in Figure 10.

This antibiotic is active against many Gram-positive as well as Gram-negative bacteria and is thus called a broad-spectrum antibiotic that inhibits β-lactamases. It inhibits all types of β-lactamases, including plasmid-mediated as well as chromosomal-intermediated [91]. Amoxicillin was resistant to *Enterococcus faecalis.* The combination with plant extracts showed no redressal of antibiotic resistance caused by *Enterococcus faecalis,* from which it can be concluded that the plant extracts were ineffective to cope with the resistance mechanism of *Enterococcus faecalis* against Amoxicillin.

#### 3.5.3. Linezolid

**Figure 11:**
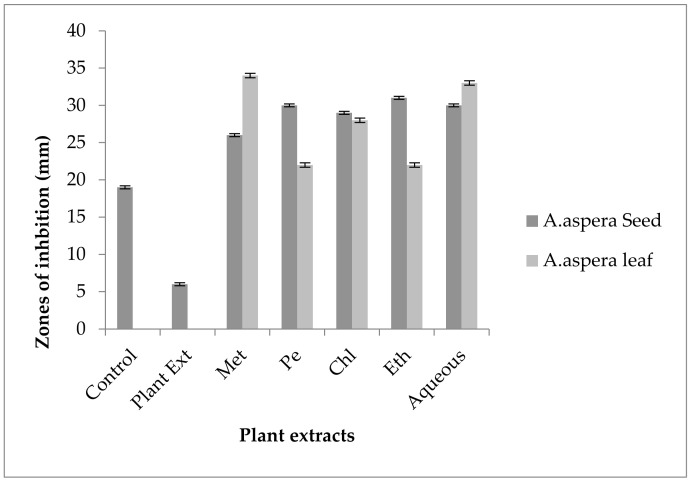
Redressal of Linezolid resistance in *Enterococcus faecalis* by using Linezolid in combination with various plant extracts of *Achyranthes aspera* (leaves and seeds).

The Linezolid inhibition zone for *Enterococcus faecalis* was 19 mm before treatment with plant extracts. The zone of inhibition for Linezolid must be equivalent to or overhead to 21 mm to be categorized as a sensitive strain. After treatment with the plant extracts of *Achyranthes aspera* seeds, the zone sizes increased. The highest zone size was measured for the ethyl acetate extract of seeds, which was 31 mm. The zone sizes for the methanolic, petroleum ether, chloroform, and aqueous extracts were 26, 30, 29, and 30 mm, respectively. No zone of inhibition was shown by any plant extract alone, as shown in Figure 11. The zone sizes increased for the *Achyranthes aspera* leaf methanolic, petroleum ether, chloroform, ethyl acetate, and aqueous extracts up to 34, 22, 28, 22, and 31 mm, respectively.

Linezolid was resistant to *Enterococcus faecalis, and* by using the above-mentioned extracts it became sensitive to the respective bacteria. The plant extracts either synergized the effect of antibiotics or re-sensitized the bacteria to the respective antibiotics. From the results, it was concluded that the plant extracts reversed the resistance mechanism of *Enterococcus faecalis* against Linezolid.

#### 3.5.4. Penicillin

**Figure 12:**
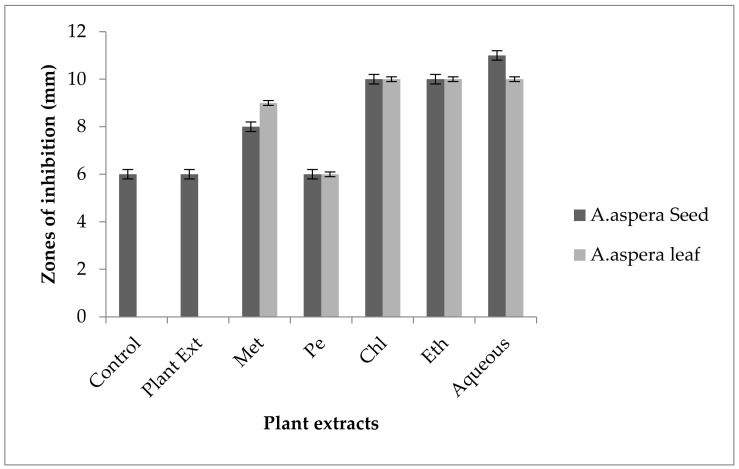
Redressal of Penicillin resistance in *Enterococcus faecalis* by using Penicillin in combination with various plant extracts of *Achyranthes aspera* (leaves and seeds).

In the case of *Enterococcus faecalis,* Penicillin showed an inhibition zone up to 8 mm before treatment with the plant extracts, as shown in Figure 12. By the standard given by CLSI (2020), the inhibition zone must be equivalent to or above 15 mm to fall in the sensitive range. After treatment with the *Achyranthes aspera* seed extracts, the zone size increased up to 11 mm by aqueous extract. The zone sizes increased up to 10 mm, by chloroform and ethyl acetate extracts of seeds, respectively, while no change in zone size was observed with methanolic and petroleum ether extracts. No zone of inhibition was shown by any plant extract alone. When the plant extracts of *Achyranthes aspera* leaves combined with the Penicillin, the highest change in zone of inhibition was observed up to 10 mm by ethyl acetate extract, chloroform, and ethyl acetate extracts, while zone sizes with methanolic increased up to 9 mm.

Through the combination of Penicillin with plant extracts, an increase in the zone size was observed, but it was not enough to fall into the sensitive category. Although the zone size observed fell into the intermediate range by many plant extracts, they cannot be considered as effective treatment method for redressal of resistance.

#### 3.5.5. Levofloxacin

**Figure 13:**
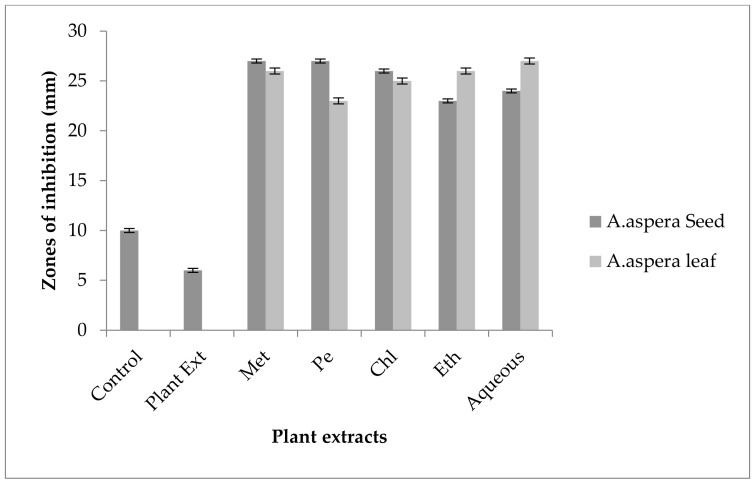
Redressal of Levofloxacin resistance in *Enterococcus faecalis* by using Levofloxacin in combination with various plant extracts of *Achyranthes aspera* (leaves and seeds).

The Levofloxacin inhibition zone for *Enterococcus faecalis* was 10 mm before treatment with plant extracts. In comparison with the standard given by CLSI (2020), the inhibition zone for Levofloxacin should be equivalent to or overhead 17 mm to be categorized as a sensitive zone. In combination with the plant extracts of *Achyranthes aspera* seeds, the zone sizes increased, and the highest zone size was measured for methanolic and petroleum ether extracts of seeds, which was 27 mm. The zone sizes for the chloroform, ethyl acetate extracts, and aqueous extracts were 26, 23, and 24 mm, respectively. No zone of inhibition was shown by any plant extract alone, as shown in Figure 13. The zone sizes increased for the *Achyranthes aspera* leaf methanolic, petroleum ether, chloroform, ethyl acetate, and aqueous extracts up to 26, 23, 25, 26, and 27 mm, respectively.

By natural plant sources, resistance can be reversed. This result suggested that with the help of plant extracts, the zone of inhibition increased to a significant level. Levofloxacin was resistant to *Enterococcus faecalis*, and by using the above-mentioned extracts, it became sensitive to the respective bacteria. The plant extracts either synergized the effect of antibiotics or re-sensitized the bacteria to the respective antibiotics.

#### 3.5.6. Vancomycin

**Figure 14:**
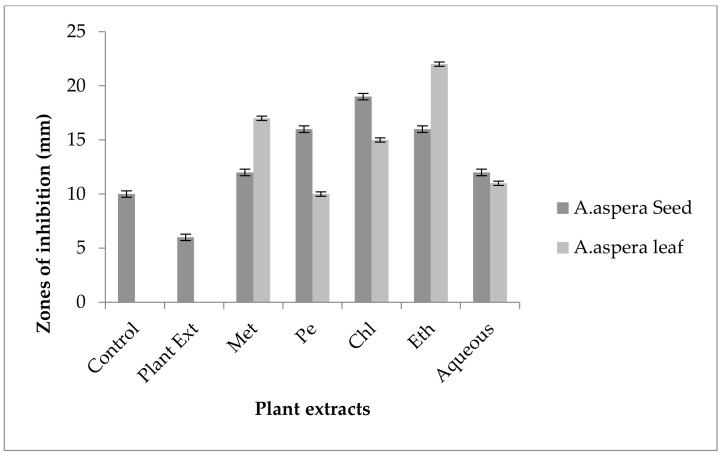
Redressal of Vancomycin resistance in *Enterococcus faecalis* by using Vancomycin in combination with various plant extracts of *Achyranthes aspera* (leaves and seeds).

In the case of *Enterococcus faecalis,* no inhibition zone was shown by any plant extract alone. The zone of inhibition of Co-trimoxazole against *Enterococcus faecalis* was up to 10 mm. To be sensitive against the respective bacteria, the zone size should be equal to or above 17 mm according to the standard table provided by the CLSI (2020). After the combination of antibiotics with the plant extract of *Achyranthes aspera* seeds, an increase in zone size was observed. The highest zone was observed with the chloroform extract of seeds, which was 19 mm. The zone sizes for the methanolic, petroleum ether, ethyl acetate, and aqueous extracts were 12, 16, 19, and 12 mm, respectively, as shown in Figure 14. *Achyranthes aspera* leaf extracts also showed an increase in the zone sizes for chloroform, ethyl acetate, and aqueous extracts up to 17, 15, 22, and 11 mm, respectively, while no change in zone size was observed with petroleum ether extract.

Vancomycin is antibactericidal, and belongs to the glycopeptide class of antibiotics which inhibit the growth of bacteria by binding to the acyl-D-ala-D-ala portion of the growing cell wall. After binding it weakens the cross-linking which is required for cell wall strength and to keep it intact [92]. The glycopeptide resistance mechanism for *Enterococcus faecalis* involves a change in the peptidoglycan synthesis pathway, which involves a change in the binding of amino acids in the specific sequence of D-ala-D-ala to either D-alanine–D-lactate or D-ala-D-serine, which leads to glycopeptide resistance [93]. Vancomycin was resistant to *Enterococcus faecalis,* and by using the above-mentioned extracts it became sensitive to the respective bacteria. The plant extracts either synergized the effect of antibiotics or re-sensitized the bacteria to the respective antibiotics. From the results, it was concluded that the plant extracts reverse the resistance mechanism of *Enterococcus faecalis* against Vancomycin.

#### 3.5.7. Imipenem

**Figure 15:**
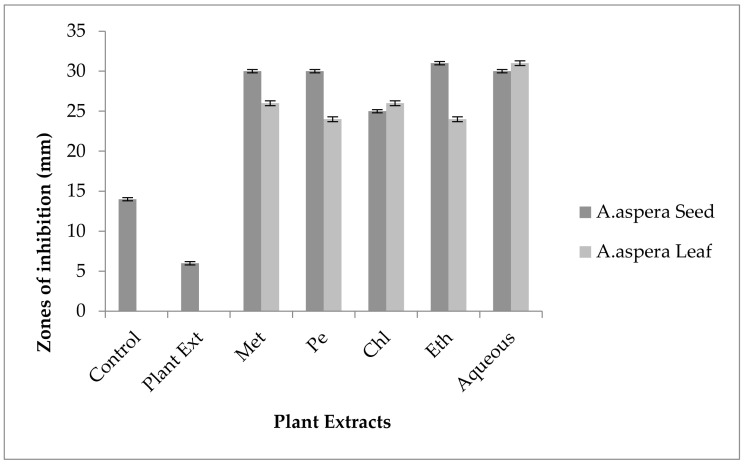
Redressal of Imipenem resistance in *Enterococcus faecalis* by using Imipenem in combination with various plant extracts of *Achyranthes aspera* (leaves and seeds).

The Imipenem zone of inhibition for *Enterococcus faecalis* was 14 mm before treatment with plant extracts. In comparison with the standard given by CLSI (2020), the inhibition zone for Imipenem should be equivalent to or overhead 21 mm to be categorized as a sensitive zone. After treating with the plant extracts of *Achyranthes aspera* seeds, the highest zone size was measured for the ethyl acetate extract of seeds, which was 31 mm. The zone sizes for the methanolic, petroleum ether, chloroform, and aqueous extracts were 30, 30, 25, and 30 mm, respectively. No zone of inhibition was shown by any plant extract alone, as shown in Figure 15. The zone sizes increased for the *Achyranthes aspera* leaf methanolic, petroleum ether, chloroform, ethyl acetate, and aqueous extracts up to 26, 24, 26, 24, and 31 mm, respectively.

Imipenem belongs to the class of antibiotics known as carbapenems. It is a broad-spectrum antibiotic with the same mechanism of action as other antibiotics belonging to the β-lactam class. It usually inhibits the synthesis of the cell wall by binding to the PBP-1 [94]. The combination with plant extracts showed a redressal of antibiotic resistance caused by *Enterococcus faecalis,* from which it can be concluded that the plantsextracts were effective to cope with the resistance mechanism of *Enterococcus faecalis* against Imipenem.

### 3.6. Antibiotic Resistant Redressal Activity for Acinetobacter baumannii

#### 3.6.1. Ciprofloxacin

**Figure 16:**
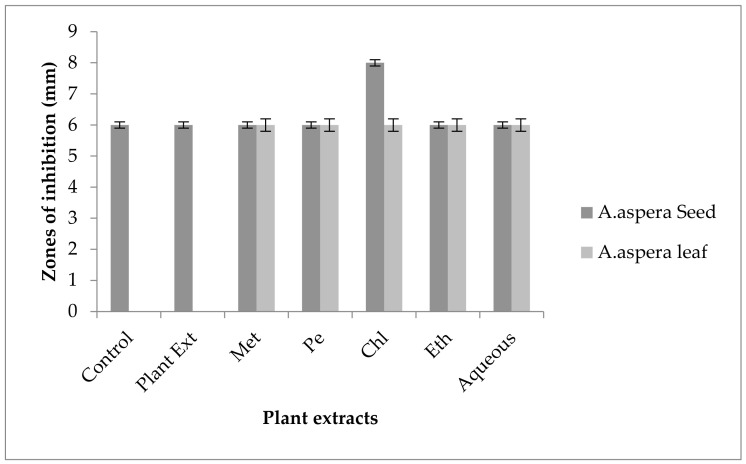
Redressal of Ciprofloxacin resistance in *Acinetobacter baumannii* by using Ciprofloxacin in combination with various plant extracts of *Achyranthes aspera* (leaves and seeds).

Ciprofloxacin showed no zones of inhibition for the *Acinetobacter baumannii* before treatment with the plant extracts. In comparison with the standard, the inhibition zone must be overhead or equivalent to 26 mm to be categorized as a sensitive zone. After the treatment with plant extracts of *Achyranthes aspera* seeds, the zone size increased up to 8 mm by the ethyl acetate extract of the seed, but it was not helpful as the antibiotic remained resistant. No change in zone size was observed with the methanolic, petroleum ether, chloroform, and aqueous extracts of any plant alone as shown in Figure 16. After the combination with the methanolic, petroleum ether, chloroform, ethyl acetate, and aqueous plant extracts of *Achyranthes aspera,* no change of zone size was observed by Ciprofloxacin as shown in Figure 16.

*Acinetobacter baumannii* showed resistance towards many antibiotics by different resistant mechanisms such as AME (Aminoglycoside-modifying enzyme), β-lactamases, target alteration, MEP (multidrug efflux pumps), and permeability defects [95]. The Ciprofloxacin resistance in *Acinetobacter baumannii* is mainly due to the efflux pumps known as H-coupled pumps. These pumps belong to MATE family [96]. From the results, it was concluded that the resistance of Ciprofloxacin against *Acinetobacter baumannii* was not reversed by any plant extract. Although a change in zone size was observed in the *Achyranthes aspera* ethyl acetate extract, it was not in the considerable range.

#### 3.6.2. Amikacin

**Figure 17:**
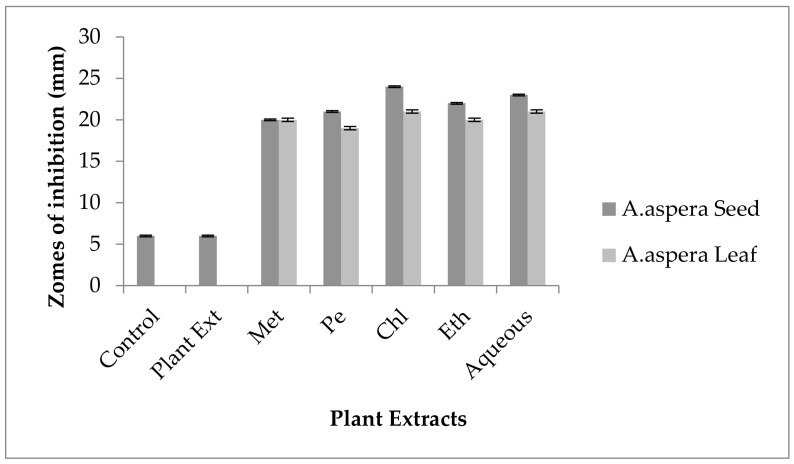
Redressal of Amikacin resistance in *Acinetobacter baumannii* by using Amikacin in combination with various plant extracts of *Achyranthes aspera* (leaves and seeds).

In the case of *Acinetobacter baumannii,* the inhibition zone for Amikacin was 18 mm without dealing with any plant extracts. The zone size of 18 mm is categorized as a sensitive zone when compared with the standard given by CLSI (2020). The AST test results after treatment with plant extracts showed an increase in the size of the inhibition zone with *Achyranthes aspera* seed extracts, which means these extracts potentiate the effect of Amikacin. The zone of inhibition with the chloroform extract of seeds was 24 mm which was the highest zone for the seed extracts. Meanwhile, the zone of inhibition for methanolic, petroleum ether, ethyl acetate, and aqueous extracts was 20, 21, 22, and 23 mm, respectively, as shown in Figure 17. No zone of inhibition was shown by any plant extract alone. After treatment with the *Achyranthes aspera* leaves, the zone size increased up to 21 mm, and with the chloroform extracts of leaves it was the highest zone of inhibition for the *Achyranthes aspera* leaves. The zone size with methanolic, petroleum ether, ethyl acetate, and aqueous extracts increased up to 20, 19, 20, and 21 mm, respectively.

The inhibition of the gene that encodes for the *ACC(6′)-Ie* enzyme led to the reversal of Amikacin resistance in many organisms [97,98]. From the above-mentioned results, it was concluded that by using plant extracts, the effect of Amikacin was enhanced. Amikacin was sensitive to the respective strain, but plant extracts potentiated the effect of Amikacin to a great extent.

#### 3.6.3. Ceftriaxone

**Figure 18:**
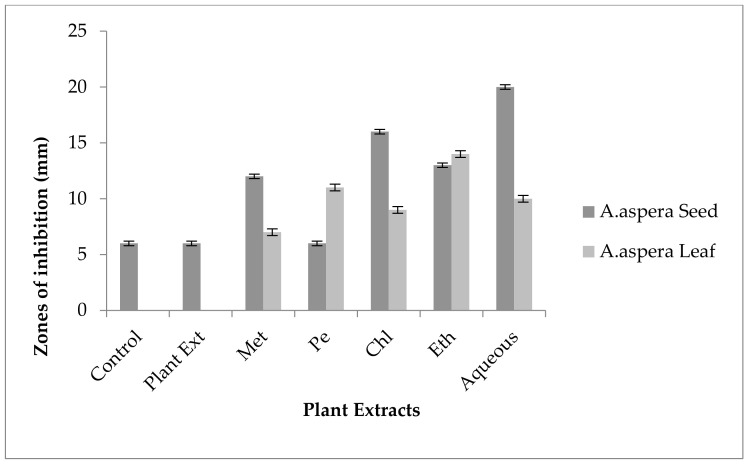
Redressal of Ceftriaxone resistance in *Acinetobacter baumannii* by using Ceftriaxone in combination with various plant extracts of *Achyranthes aspera* (leaves and seeds).

Ceftriaxone showed no zone of inhibition against *Acinetobacter baumannii* without handling the plant extracts, as shown in Figure 18. After comparison with the standard zone values given by CLSI (2020), the inhibition zones must be equivalent to or overhead 23 mm to fall in the sensitive range. After treatment with the *Achyranthes aspera* seed extracts, the zone size increased up to 20 mm by the aqueous extract, which lay in the intermediate zone range. The zone sizes with other extracts of seeds increases up to 12, 16, and 13 mm by methanolic, chloroform, and ethyl acetate extract, respectively. Meanwhile, no change in zone size was observed with the petroleum ether extract of *Achyranthes aspera* seeds. No zone of inhibition was shown by any plant extract alone. When the plant extracts of *Achyranthes aspera* leaves combined with Ceftriaxone, the highest change in the zone of inhibition was observed up to 14 mm ethyl acetate extracts. Meanwhile, the zone sizes with methanolic, petroleum ether, chloroform, and aqueous extracts increased up to 7, 11, 9, and 10 mm, respectively.

Ceftriaxone belongs to the family of antibiotics known as cephalosporin. It is a third-generation antibiotic acting as the cell wall synthesis inhibitor. It inhibits the cell wall synthesis irreversibly by binding to the PBPs (Penicillin-binding proteins) called transpeptidases [99]. Resistance to Ceftriaxone in *Acinetobacter baumannii* develops due to the CTX-M-2, which is an ESBL. It increases the hydrolysis of Ceftriaxone which leads to its ineffectiveness [100]. By the combination of Ceftriaxone with plant extracts, an increase in the zone size was observed, but it was not enough to fall into the sensitive category. Although the zone size observed fell into the intermediate range by many plant extracts, they could not be considered as an effective treatment method for the redressal of resistance.

#### 3.6.4. Levofloxacin

**Figure 19:**
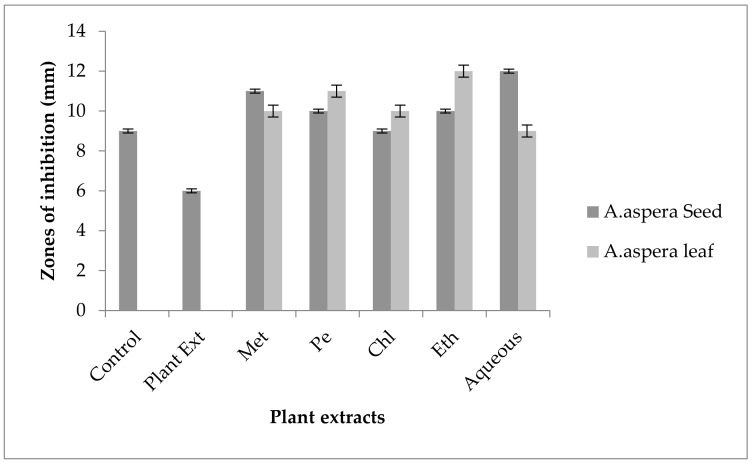
Redressal of Levofloxacin resistance in *Acinetobacter baumannii* by using Levofloxacin in combination with various plant extracts of *Achyranthes aspera* (leaves and seeds).

The Levofloxacin zone of inhibition for *Acinetobacter baumannii* was 9 mm before treatment with plant extracts. According to the standard table given by CLSI (2020) the zone of inhibition for Levofloxacin should be equal to or above 21 mm to be categorized as sensitive. After treatment with the plant extracts of *Achyranthes aspera* seeds, the zone sizes increased somehow but they remained resistant. The highest zone size was measured for the aqueous extract of seed which was 12 mm. The zones sizes for the methanolic, petroleum ether, chloroform, and ethyl acetate extracts were 11, 10, 9, and 10 mm, respectively. No zone of inhibition was shown by any plant extract alone as shown in Figure 19. The zone sizes increases for the *Achyranthes aspera* leaves methanolic, petroleum ether, chloroform, ethyl acetate and aqueous extracts up to 10, 11, 10, 12, and 9 mm, respectively.

The Levofloxacin resistance in *Acinetobacter baumannii* is mainly due to the efflux pumps known as H-coupled pumps. These pumps belong to the MATE family [96]. From the results, it was concluded that the resistance of Levofloxacin against *Acinetobacter baumannii* was not reversed by any plant extract. Although a change in zone sizes was observed by some plant extracts, it was not in the considerable range.

#### 3.6.5. Ceftazidime

**Figure 20:**
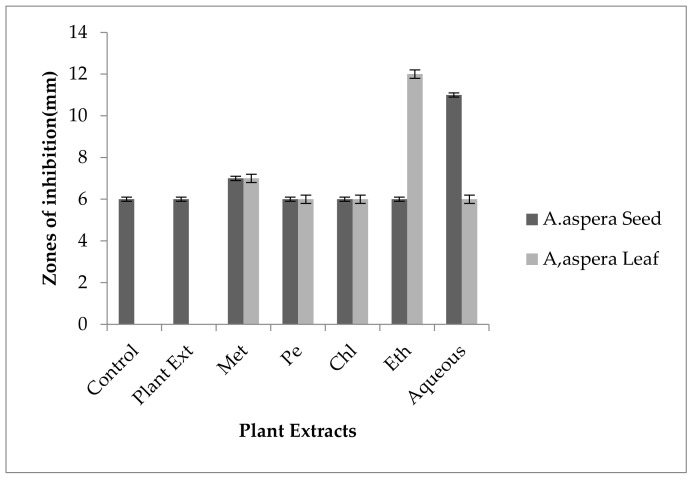
Redressal of Ceftazidime resistance in *Acinetobacter baumannii* by using Ceftazidime in combination with various plant extracts of *Achyranthes aspera* (leaves and seeds).

Ceftazidime showed no zone of inhibition before treatment with plant extracts for *Acinetobacter baumannii.* The inhibition zone must be equivalent to or overhead 21 mm when compared with the standard given by CLSI (2020). No zone of inhibition was shown by any plant extract alone. *Achyranthes aspera* seed extracts such as methanolic, petroleum ether, chloroform, ethyl acetate, and aqueous extracts were used in combination with the Ceftazidime but no change in zone was observed as shown in Figure 20. With the combination of Ceftazidime to *Achyranthes aspera* leaf extracts no change in the zone sizes was observed.

Ceftazidime belongs to the family of antibiotics known as cephalosporin. It is a third-generation antibiotic acting as the cell wall synthesis inhibitor. It inhibits the cell wall synthesis irreversibly by binding to the PBPs (Penicillin-binding proteins) called as transpeptidases [99]. An ESBL known as PER-1 is the main cause of resistance in Cephalosporin, especially in the extended spectrum such as Ceftazidime [101]. The resistance in *Acinetobacter baumannii* against Ceftazidime was developed by the production of enzymes. The combination with plant extracts showed no redressal of antibiotic resistance caused by *Acinetobacter baumannii.* Thus, it can be concluded that the plants extracts were ineffective to cope up with the resistance mechanism of *Acinetobacter baumannii* against Ceftazidime.

#### 3.6.6. Imipenem

**Figure 21:**
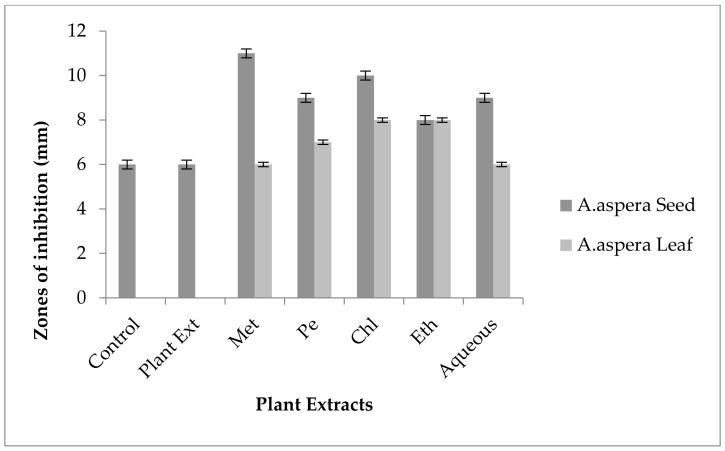
Redressal of Imipenem resistance in *Acinetobacter baumannii* by using Imipenem in combination with various plant extracts of *Achyranthes aspera* (leaves and seeds).

No zone of inhibition was shown by any plant extract alone for *Acinetobacter baumannii,* and no zone of inhibition of Imipenem was observed against respective bacteria before combination with the plant extracts. The zone size for the Imipenem should be equal to or above 23 mm to be called sensitive against the respective bacterial strains, according to the CLSI (2020). No change in zone size was observed when treated with the plant extracts of *Achyranthes aspera* seeds, as shown in Figure 21. Methanolic, petroleum ether, chloroform, ethyl acetate, and aqueous extracts of *Achyranthes aspera* leaves showed no activity either alone or in combination with the antibiotic.

The resistance of Imipenem in *Acinetobacter baumannii* is mainly due to the action of efflux pumps action [102]. The resistance in *Acinetobacter baumannii* against Imipenem was developed by the action of efflux pumps. The combination with plant extracts showed no redressal of antibiotic resistance caused by *Acinetobacter baumannii.* Thus, it can be concluded that the plants extracts were ineffective to cope up with the resistance mechanism of *Acinetobacter baumannii* against Imipenem.

#### 3.6.7. Co-trimoxazole

**Figure 22:**
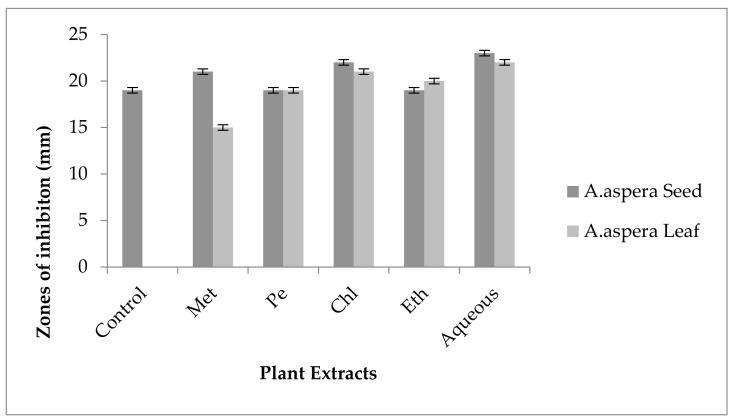
Redressal of Co-trimoxazole resistance in *Acinetobacter baumannii* by using Co-trimoxazole in combination with various plant extracts of *Achyranthes aspera* (leaves and seeds).

For *Acinetobacter baumannii,* no zone of inhibition was shown by any plant extract alone. The zone of inhibition of Co-trimoxazole for *Acinetobacter baumannii* was 19 mm. To be sensitive against the respective bacteria, the zone size should be equal to or above 16 mm according to the standard table provide by CLSI (2020). The combination of antibiotics with the plant extract of *Achyranthes aspera* seeds resulted in an increase in the zone size. The highest zone was observed with the chloroform extract of seeds which is 22 mm. The zone sizes for the methanolic, petroleum ether, ethyl acetate and aqueous extracts were 21, 16, 19, and 23 mm, as shown in Figure 22. *Achyranthes aspera* leaf extracts also showed an increase in the zone sizes for chloroform, ethyl acetate, and aqueous extracts up to 21, 22, and 20 mm, respectively. Meanwhile, in the combination with methanolic and petroleum ether extracts, no change in zone size was observed.

Co-trimoxazole belongs to the sulfonamide group of antibiotics. They perform their function by inhibiting the synthesis of folate by directly acting as competitor of PABA (p-aminobenzoic acid). This antibiotic is actually a combination of Trimethoprim and Sulfamethoxazole [88]. The resistance mechanism of *Acinetobacter baumannii* against Co-trimoxazole is the active efflux pumps known as *AdeABC* that lead to the inhibition of the entry of drugs into the bacterial cell [103]. From the above-mentioned results, it was concluded that by using plant extracts, the effect of Co-trimoxazole was enhanced. Co-trimoxazole was sensitive to the respective strain but plant extracts potentiated the effect of Co-trimoxazole to a great extent.

### 3.7. Antibiotic Resistant Redressal Activity for Klebsiella pneumoniae

#### 3.7.1. Ciprofloxacin

**Figure 23:**
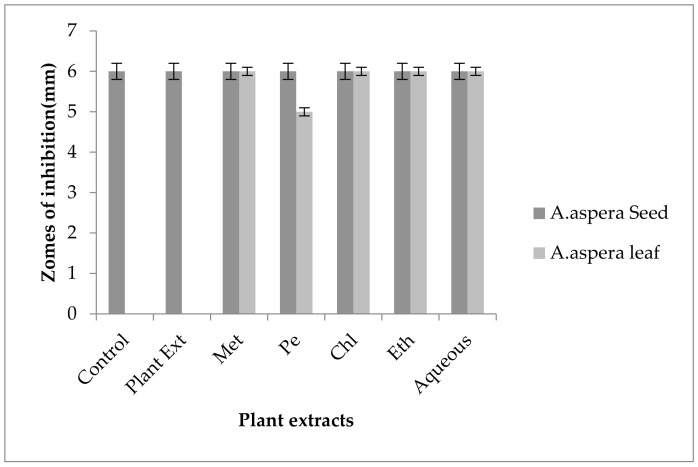
Redressal of Ciprofloxacin resistance in *Klebsiella pneumoniae* by using Ciprofloxacin in combination with various plant extracts of *Achyranthes aspera* (leaves and seeds).

Ciprofloxacin showed no zones of inhibition for the *Klebsiella pneumoniae* before treatment with the plant extracts. By the standard given by CLSI (2020), it was concluded that the inhibition zone must be overhead or equivalent to 26 mm to be concluded as sensitive zone. No change in zone size was observed with methanolic, petroleum ether, chloroform, and aqueous extracts of any plant alone as shown in Figure 23. After combination with the methanolic, petroleum ether, chloroform, ethyl acetate, and aqueous plant extracts of *Achyranthes aspera* seeds, no change of zone size was observed by Ciprofloxacin.

ESBLs (extended-spectrum β-Lactamases) and cabapenemases production are the two main mechanisms for the development of resistance in *Klebsiella pneumoniae* [104]. Ciprofloxacin resistance to *Klebsiella pneumoniae* became resistant through the modification of the *gyrA* gene after the mutation could be reduced by knocking out the following genes: *recA, xseA, fis, recC,* and *tolC* [105]. From the results, it was concluded that the plant extracts are not effective for redressal of the respective antibiotic resistance in bacteria. EPIs are needed for the reversal of antibiotic resistance in Ciprofloxacin.

#### 3.7.2. Amikacin

**Figure 24:**
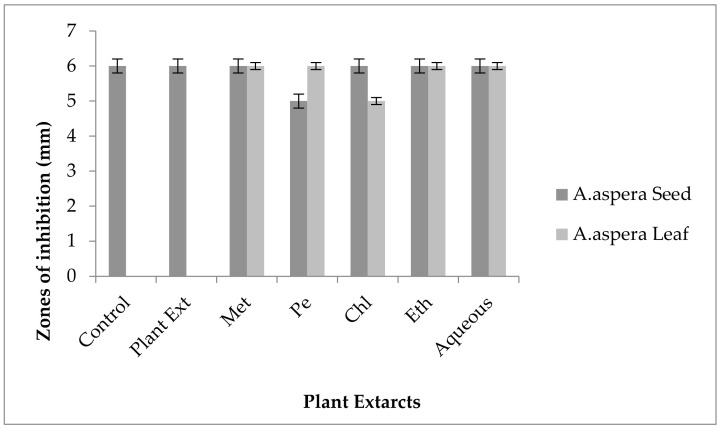
Redressal of Amikacin resistance in *Klebsiella pneumoniae* by using Amikacin in combination with various plant extracts of *Achyranthes aspera* (leaves and seeds).

For *Klebsiella pneumoniae,* no zone of inhibition was shown by any plant extract alone. The zone of inhibition of Amikacin for *Klebsiella pneumoniae* was 19 mm, and to be sensitive against the respective bacteria the zone size must be equivalent to or overhead 16 mm according to the standard table provide by the CLSI (2020). The combination of antibiotics with the plant extract of *Achyranthes aspera* seed extracts did not change at all and increased in size, which meant these extracts did not have an increased effect of Amikacin, as shown in Figure 24. No zone of inhibition was shown by any plant extract alone. Methanolic, petroleum ether, chloroform, ethyl acetate, and aqueous extracts of *Achyranthes aspera* leaves showed no activity either alone or in combination with the antibiotic. AME (Aminoglycoside-modifying enzyme) inhibition is needed for reversing the resistance of aminoglycosides [106]. From the present study results, it was concluded that the used plant extracts were unable to reverse the antibiotic resistance in the respective bacteria. For the redressal of resistance in Amikacin acetylation, encoding gene inhibition is needed.

#### 3.7.3. Ceftriaxone

**Figure 25:**
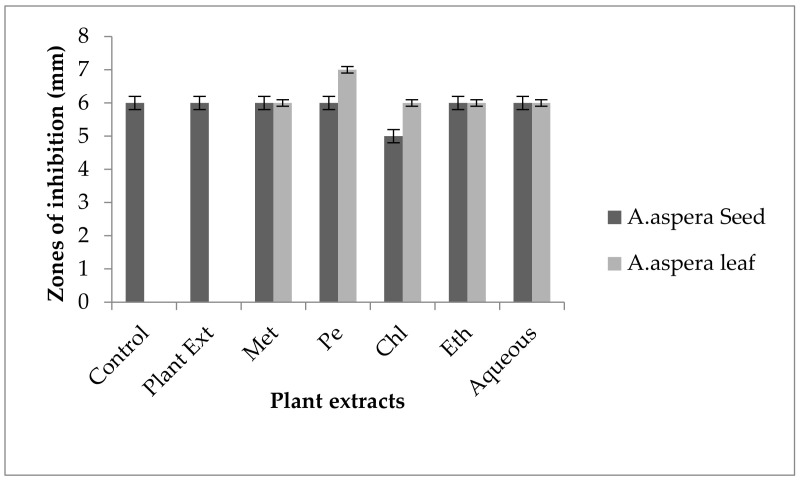
Redressal of Ceftriaxone resistance in *Klebsiella pneumonia* by using Ceftriaxone in combination with various plant extracts of *Achyranthes aspera* (leaves and seeds).

Ceftriaxone showed no inhibition zone before be handled with plant extracts for *Klebsiella pneumoniae.* The inhibition zone must be equivalent to or overhead 21 mm when compared to standard given by CLSI (2020). No inhibition zone was shown by any plant extract alone. *Achyranthes aspera* seed and leaf extracts such as methanolic, petroleum ether, chloroform, ethyl acetate, and aqueous extracts were used in combination with the Ceftriaxone but no change in zone was observed as shown in Figure 25.

Ceftriaxone is used for SSI treatment in *Klebsiella pneumonia* [107]. The resistance of *Klebsiella pneumoniae* to Ceftriaxone is mainly due to the production of ESBLs [108]. The combination with plant extracts showed no redressal of antibiotic resistance caused by *Klebsiella pneumoniae.* Thus, it can be concluded that the plants extracts were ineffective to cope up with the resistance mechanism of *Klebsiella pneumoniae* against Ceftriaxone.

#### 3.7.4. Levofloxacin

**Figure 26:**
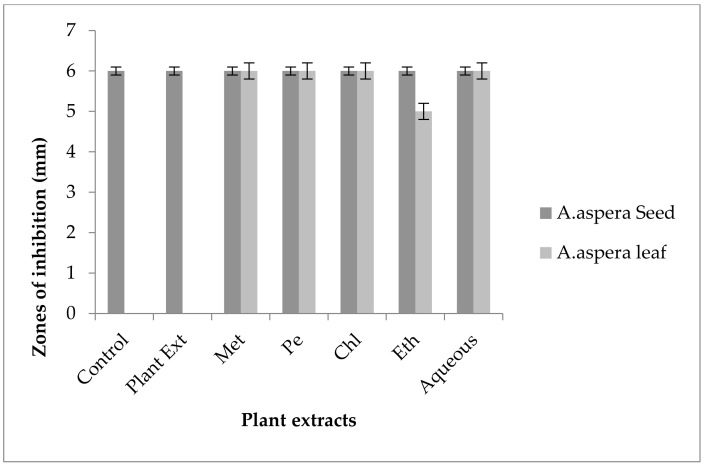
Redressal of Levofloxacin resistance in *Klebsiella pneumoniae* by using Levofloxacin in combination with various plant extracts of *Achyranthes aspera* (leaves and seeds).

No inhibition zone was shown by any plant extract alone for *Klebsiella pneumoniae,* and no zone of inhibition of Levofloxacin was observed against the respective bacteria before the combination with plant extracts. The zone size for the Levofloxacin should be equal to or above 21 mm to be called sensitive against respective bacterial strain according to CLSI (2020). No change in zone size was observed when treated with the plant extracts of *Achyranthes aspera* seeds and leaves as shown in Figure 26.

Levofloxacin is resistant to *Klebsiella pneumoniae*, and became resistant to the modification of the *gyrA* gene after the mutation was reduced by knocking out the following genes: *recA, xseA, fis, recC,* and *tolC* [105]. From the results, it was concluded that the plant extracts were not effective for redressal of the respective antibiotic resistance in bacteria.

#### 3.7.5. Imipenem

**Figure 27:**
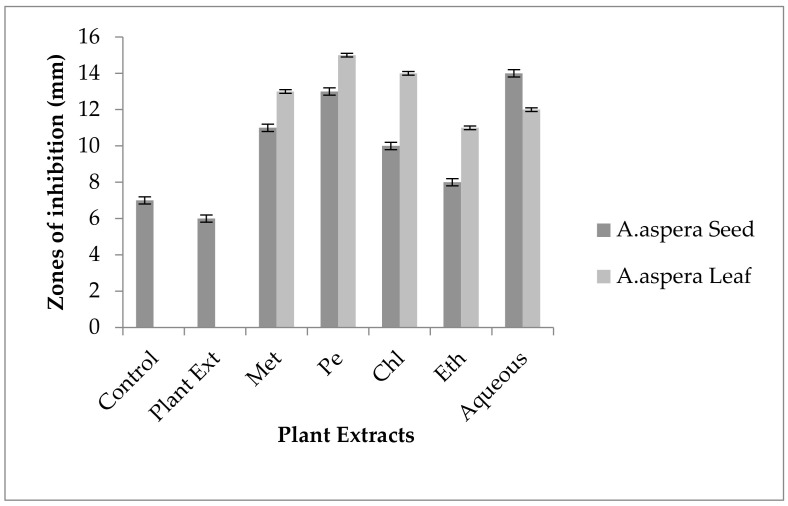
Redressal of Imipenem resistance in *Klebsiella pneumoniae* by using Imipenem in combination with various plant extracts of *Achyranthes aspera* (leaves and seeds).

The Imipenem zone of inhibition for *Klebsiella pneumoniae* was 7 mm before treatment with plant extracts. According to the standard table given by CLSI (2020), the zone of inhibition for Imipenem should be equal to or above 23 mm to be categorized as sensitive. After treatment with the plant extracts of *Achyranthes aspera* seeds, the zone sizes increased somehow but they remained resistant. The highest zone size was measured for the aqueous extract of seed which was 14 mm. The zones sizes for the methanolic, petroleum ether, chloroform, ethyl acetate extracts were 11, 13, 10, and 8 mm, respectively. No zone of inhibition was shown by any plant extract alone, as shown in Figure 27. The zone sizes increased for the *Achyranthes aspera* leaves methanolic, petroleum ether, chloroform, ethyl acetate, and aqueous extracts up to 13, 15, 14, 11, and 12 mm, respectively.

Imipenem is resistant to *Klebsiella pneumoniae* due to the production of Carbapenemases [109]. From the results, it was concluded that the plant extracts were not effective in the redressal of antibiotic resistance against respective bacteria. Although an increase in zone sizes was observed by various plant extracts, they were unable to be recorded as sensitive zones.

#### 3.7.6. Ceftazidime

**Figure 28:**
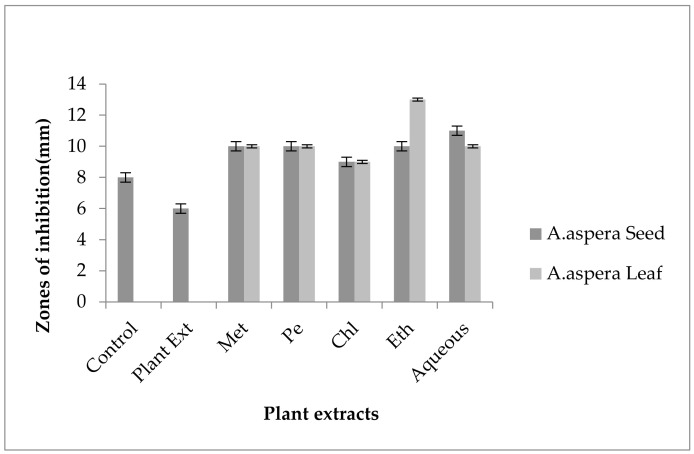
Redressal of Ceftazidime resistance in *Klebsiella pneumoniae* by using Ceftazidime in combination with various plant extracts of *Achyranthes aspera* (leaves and seeds).

In the case of *Klebsiella pneumoniae,* the Ceftazidime showed an inhibition zone up to 8 mm before treatment with the plant extracts, as shown in Figure 28. In comparison with the standard given by CLSI (2020), the inhibition zone must be equivalent to or overhead 21 mm to fall in the sensitive range. After treatment with the *Achyranthes aspera* seed extracts, the zone size increased up to 11 mm by aqueous extract. The zone sizes with other extracts of seeds increased up to 10, 10, 9, and 10 mm by methanolic, petroleum ether, chloroform, and ethyl acetate extracts, respectively. No zone of inhibition was shown by any plant extract alone. When the plant extracts of *Achyranthes aspera* leaves combined with the Ceftazidime, the highest change in the zone of inhibition was observed up to 13 mm by ethyl acetate extract. Meanwhile, zone sizes with methanolic, petroleum ether, chloroform and aqueous extracts increased up to 10, 10, 9, and 10 mm, respectively.

Ceftazidime is used for SSI (surgical site infections) treatment caused by *Klebsiella pneumoniae* [107]. The resistance of *Klebsiella pneumoniae* to Ceftazidime is mainly due to the production of ESBLs [108]. The combination with plant extracts showed no redressal of antibiotic resistance caused by *Klebsiella pneumoniae,* although an increase in zone sizes was observed but still fell in the resistant category. Thus, it can be concluded that the plant extracts were ineffective to cope with the resistance mechanism of *Klebsiella pneumoniae* against Ceftazidime.

#### 3.7.7. Co-trimoxazole

**Figure 29:**
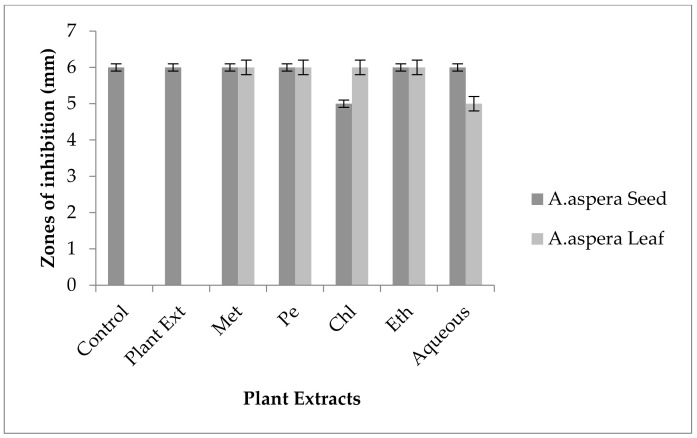
Redressal of Co-trimoxazole resistance in *Klebsiella pneumonia* by using Co-trimoxazole in combination with various plant extracts of *Achyranthes aspera* (leaves and seeds).

For *Klebsiella pneumoniae,* no inhibition zone was shown by any plant extract alone. No zone of inhibition was shown by Co-trimoxazole against *Klebsiella pneumoniae.* To be sensitive against the respective bacteria, the zone size should be equal to or above 16 mm according to the standard table provide by CLSI (2020). The combination of antibiotics with the plant extracts of *Achyranthes aspera* seeds and leaves provided no results as no zone of inhibition was observed, as shown in Figure 29. The resistance of Co-trimoxazole develops in *Klebsiella pneumoniae* mainly due to the modification of target site [110]. Co-trimoxazole resistance for the respective bacteria could not be reversed by the combination with the plant extracts.

### 3.8. Antibiotic Resistant Redressal Activity for Pseudomonas aeruginosa

#### 3.8.1. Ciprofloxacin

**Figure 30:**
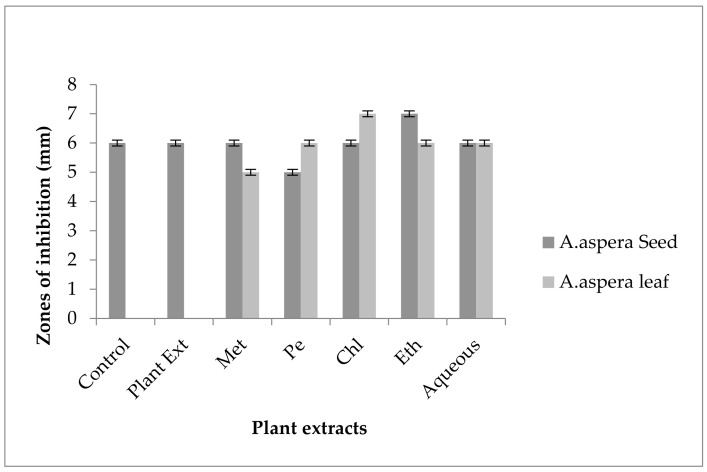
Redressal of Ciprofloxacin resistance in *Pseudomonas aeruginosa* by using Ciprofloxacin in combination with various plant extracts of *Achyranthes aspera* (leaves and seeds).

Ciprofloxacin showed no zones of inhibition for the *Pseudomonas aeruginosa* before treatment with the plant extracts. In comparison with the standard, the inhibition zone must be overhead or equivalent to 26 mm to be reflected as sensitive. No change in zone size was observed with methanolic, petroleum ether, chloroform, or aqueous extracts of any plant alone, as shown in Figure 30. After the combination with the methanolic, petroleum ether, chloroform, ethyl acetate, and aqueous plant extracts of *Achyranthes aspera* seeds and leaves, no change of zone size was observed by Ciprofloxacin.

The activation of the efflux pumps led to the development of resistance in *Pseudomonas aeruginosa* [79]. The resistance to Ciprofloxacin in bacteria was acquired mainly by two mechanisms: One was the modification of target site and the other was by the activation of efflux pumps. Target site modification is done by the modification of *gyrAB* as well as *parCE* by which the affinity of Ciprofloxacin decreased for DNA gyrase and enzyme topoisomerase IV. The activation of the efflux pumps inhibited the entry of antibiotics into the bacterial cell [111]. The resistance to Ciprofloxacin can be reduced by twofolds by the inhibition of *SbmC* genes which were discovered by the target-mediated sRNA [109]. In the present study, no change in zone size was measured. From the results, it was concluded that plant extracts were ineffective for the redressal of antibiotic resistance.

#### 3.8.2. Amikacin

**Figure 31:**
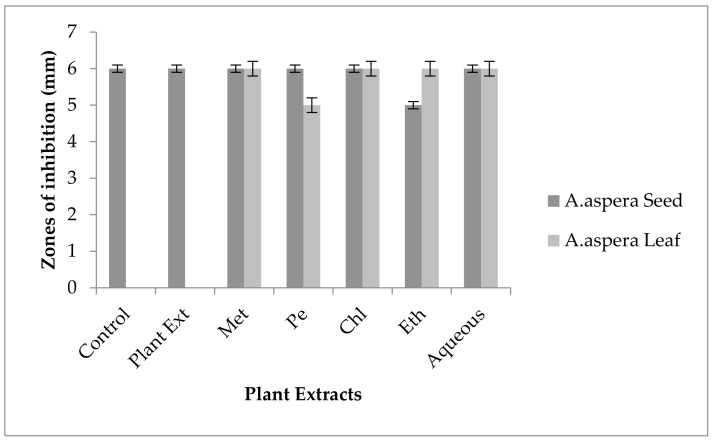
Redressal of Amikacin resistance in *Pseudomonas aeruginosa* by using Amikacin in combination with various plant extracts of *Achyranthes aspera* (leaves and seeds).

In the case of *Pseudomonas aeruginosa,* no zone of inhibition was shown by any plant extract alone. The inhibition zone of Amikacin for *Pseudomonas aeruginosa* was none. To be sensitive against the respective bacteria, the zone size must be equivalent to or overhead 18 mm according to the standard table provide by CLSI (2020). After dealing with plant extracts, AST results indicated that the zone of inhibition with *Achyranthes aspera* seed and leaf extracts did not change at all, and increased in size, which meant that these extracts did not have an increased effect of Amikacin, as shown in Figure 31. No zone of inhibition was shown by any plant extract alone.

The resistant mechanism for aminoglycoside in *Pseudomonas aeruginosa* is active efflux pumps [80]. In the present study, no change in zone size was measured. From the results, it was concluded that plant extracts were ineffective for the redressal of antibiotic resistance.

#### 3.8.3. Ceftriaxone

**Figure 32:**
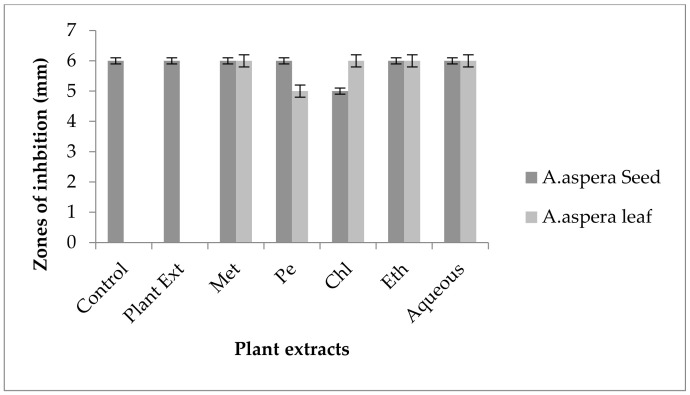
Redressal of Ceftriaxone resistance in *Pseudomonas aeruginosa* by using Ceftriaxone in combination with various plant extracts of *Achyranthes aspera* (leaves and seeds).

Ceftriaxone showed no inhibition zone before treatment with the plant extracts for *Pseudomonas aeruginosa.* The inhibition zone must be equivalent to or overhead 21 mm when compared with the standard given by CLSI (2020). No inhibition zone was shown by any plant extract alone. *Achyranthes aspera* seed extracts such as methanolic, petroleum ether, chloroform, ethyl acetate, and aqueous extracts were used in combination with Ceftriaxone, but no change in zone was observed, as shown in Figure 32. With the combination of Ceftriaxone with *Achyranthes aspera* leaf extracts, no change in the zone sizes was measured. In the present study, no change in zone size was measured. From the results, it was concluded that plant extracts were ineffective for the redressal of antibiotic resistance.

#### 3.8.4. Levofloxacin

**Figure 33:**
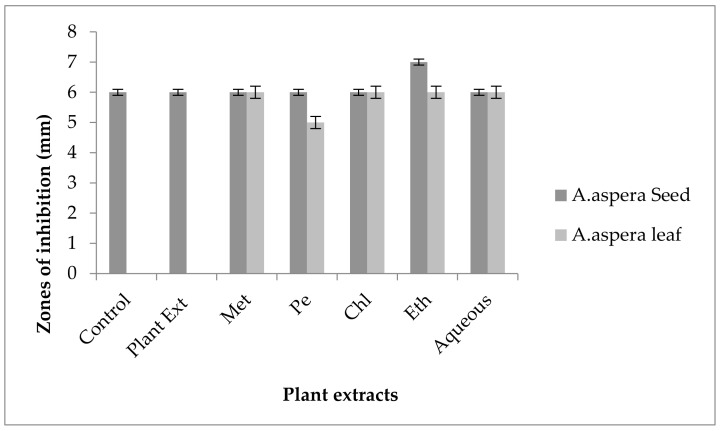
Redressal of Levofloxacin resistance in *Pseudomonas aeruginosa* by using Levofloxacin in combination with various plant extracts of *Achyranthes aspera* (leaves and seeds).

No inhibition zone was shown by any plant extract alone against *Pseudomonas aeruginosa,* and no inhibition zone of Levofloxacin was observed against respective bacteria before combination with plant extracts. The zone size for the Levofloxacin should be equal to or above 21 mm to be called sensitive against the respective bacterial strain according to CLSI (2020). No change in zone size was observed when treated with the plant extracts of *Achyranthes aspera* seeds and leaves as shown in Figure 33. The activation of the efflux pumps led to the development of resistance in *Pseudomonas aeruginosa* [111]. The activation of efflux pumps inhibited the entry of antibiotic into the bacterial cell [79]. In the present study, no change in zone size was measured. From the results, it was concluded that plant extracts were ineffective for redressal of antibiotic resistance.

#### 3.8.5. Imipenem

**Figure 34:**
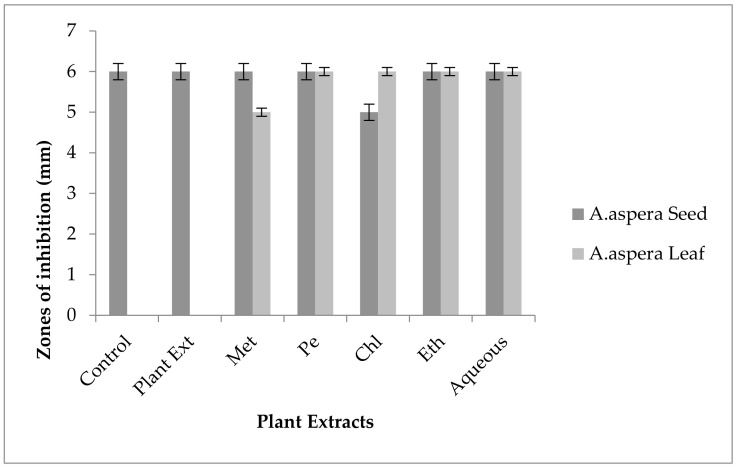
Redressal of Imipenem resistance in *Pseudomonas aeruginosa* by using Imipenem in combination with various plant extracts of *Achyranthes aspera* (leaves and seeds).

No inhibition zone was observed by plant extract alone against *Pseudomonas aeruginosa,* and no inhibition zone of Imipenem was seen against the respective bacteria before combination with plant extracts. The zone size for the Imipenem should be equal to or above 23 mm to be called sensitive against the respective bacterial strains according to CLSI (2020). No change in zone size was observed when treated with the plant extracts of *Achyranthes aspera* seeds and leaves, as shown in Figure 34. *OprD* is a known porin frequently involved in the resistance process of Imipenem in *Pseudomonas aeruginosa* [111]. From the results, it was concluded that plant extracts were ineffective for redressal of antibiotic resistance.

#### 3.8.6. Co-trimoxazole

**Figure 35:**
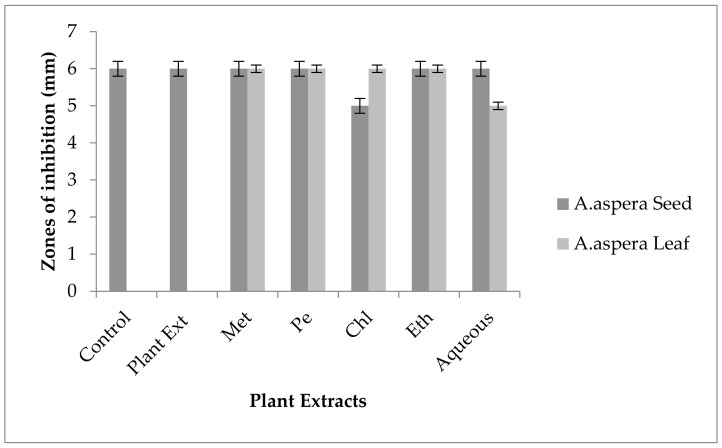
Redressal of Co-trimoxazole resistance in *Pseudomonas aeruginosa* by using Co-trimoxazole in combination with various plant extracts of *Achyranthes aspera* (leaves and seeds).

For *Pseudomonas aeruginosa,* no zone of inhibition was shown by any plant extract alone. No zone of inhibition was shown by Co-trimoxazole against *Pseudomonas aeruginosa.* To be sensitive against respective bacteria, the zone size should be equal to or above 16 mm according to the standard table provide by the CLSI (2020). The combination of antibiotics with the plant extracts of *Achyranthes aspera* seeds and leaves provided no results as no zone of inhibition was observed, as shown in Figure 35. From the results, it was concluded that plant extracts were ineffective for redressal of antibiotic resistance.

#### 3.8.7. Ceftazidime

**Figure 36:**
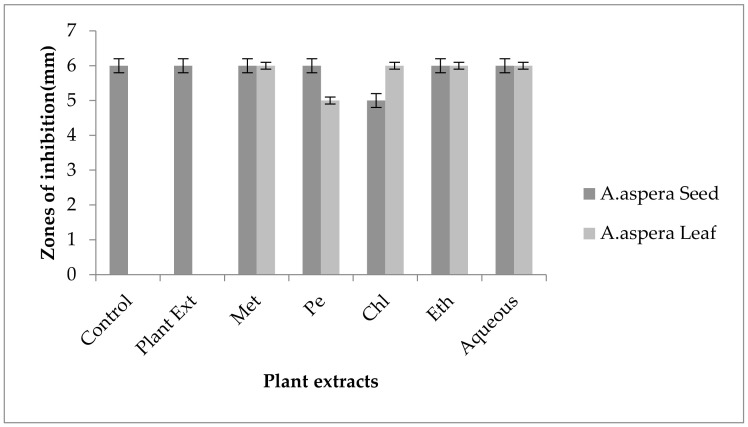
Redressal of Ceftazidime resistance in *Pseudomonas aeruginosa* by using Ceftazidime in combination with various plant extracts of *Achyranthes aspera* (leaves and seeds).

Ceftazidime showed no zones of inhibition for the *Pseudomonas aeruginosa* before treatment with the plant extracts. In comparison with the standard, the inhibition zone must be equivalent to or overhead 26 mm to be reflected as sensitive. No change in zone size was observed with methanolic, petroleum ether, chloroform, and aqueous extracts of any plant alone, as shown in Figure 36. After combination with the methanolic, petroleum ether, chloroform, ethyl acetate, and aqueous plant extracts of *Achyranthes aspera* seeds and leaves, in the present study, no change in zone size was measured. From the results, it was concluded that plant extracts were ineffective for redressal of antibiotic resistance.

## 4. Conclusions

One way toward the acceleration of antibiotic drug discovery and expansion processes is by redressal of antibiotic resistance of currently available antibiotics by co-administering these antibiotics with resistance breakers or antibiotic adjuvants. Plants contain secondary metabolites such as alkaloids, tannins, and polyphenols, and these compounds play an important role as resistance breakers, as well as antimicrobial agents. In the present study, phytochemical analysis was performed for each plant extract for the determination of bioactive compounds. According to the WHO, Methicillin-resistant *Staphylococcus aureus* and VRE (Vancomycin-resistant *Enterococci*) are in high need of new antimicrobial agents. Plant extracts provided positive results for the redressal of antibiotic resistance of these two bacteria. For *MRSA*, plant extracts provided reversal activity from resistant to sensitive for Cefoxitin, Penicillin, and Co-trimoxazole, while results were from resistant to intermediate for Amikacin and Levofloxacin. For VRE, plant extracts provided reversal activity for CIP, LEV, LZD, IMP, and VA. Plant extracts can be used for the redressal of antibiotic resistance, and this can help us to use old antibiotics and discover new components that can act as new antimicrobial agents.

## Figures and Tables

**Table 1:** Phytochemical screening of *Achyranthes aspera* seed extracts.

Tests for	Extracts of *Achyranthes aspera* (Seeds)				
	Methanolic Extract	Petroleum Ether Extract	Chloroform Extract	Ethyl Acetate Extract	Aqueous Extract
Alkaloids					
• Wagner’s test	++	-	-	+++	-
• Mayer’s test	+	-	-	++	-
• Tannic acid test	-	-	++	-	++
Carbohydrates		-			
• Barfoed’s test	-	-	-	++	-
• Fehling test	+	-	++	+++	+
• Molish test	++	++	++	++	++
Glycosides					
• Cardiac test	-	+++	+++	++	-
• Legal’s test	-	++	+++	-	++
Proteins and amino acids					
• Biuret test	-	-	-	-	-
• Ninhydrin test	-	+++	-	-	++
Fixed oils and fat					
• Spot test	-	-	-	+++	+++
Phenolics					
• Ferric chloride test	+	-	-	++	+
Tannins					
• Gelatin test	++	-	-	-	-
Terpenes	++	++	+	++	+++
Flavonoids	-	-	-	-	-
Saponins	++	-	+++	++	-
Steroids					
• Liberman–Burchard reaction	++	+++	+++	+++	++
Anthraquinones	-	-	-	-	-
Coumarin	+++	++	++	+++	+
Phlobatannins	+++	-	-	-	-
Emodins	-	-	-	-	-
Anthocyanins	-	-	-	-	-

High concentration (+++), moderate concentration (++), low concentration (+), absence (-).

**Table 2:** Phytochemical screening of *Achyranthes aspera* leaf extracts.

Tests for	Extracts of *Achyranthes aspera* (Leaves)				
	Methanolic Extract	Petroleum Ether Extract	Chloroform Extract	Ethyl Acetate Extract	Aqueous Extract
Alkaloids					
• Wagner’s test	-	++	-	-	-
• Mayer’s test	-	-	-	-	-
• Tannic acid test	+	++	++	-	-
Carbohydrates					
• Barfoed’s test	-	-	-	-	-
• Fehling test	++	+	+++	+	+++
• Molish test	-	-	++	++	-
Glycosides					
• Cardiac test	-	-	-	+++	++
• Legal’s test	-	-	-	+++	-
Proteins and amino acids					
• Biuret test	-	-	-	-	-
• Ninhydrin test	-	-	-	-	-
Fixed oils and fat					
• Spot test	++	+++	-	-	+
Phenolics					
• Ferric chloride test	++	-	++	++	++
Tannins					
• Gelatin test	++	-	-	-	-
Terpenes	++	+	++	+++	+++
Flavonoids	-	-	-	-	-
Saponins	+++	-	-	-	++
Steroids					
• Liberman–Burchard reaction	-	-	-	++	++
Anthraquinones	-	-	-	-	-
Coumarins	-	++	++	+++	+
Phlobatannins	-	-	-	-	-
Emodins	-	-	-	-	-
Anthocyanins	-	-	-	-	-

High concentration (+++), moderate concentration (++), low concentration (+), absence (-).

**Table 3:** *MRSA* AST (antibiotic susceptibility tests) results in comparison with the standard guidelines of CLSI (Clinical Laboratory Standard Institute 2020).

SR#	Antibiotics Used for *Staphylococcus aureus*	Zones of Inhibition for *Staphylococcus aureus* (mm)	Zones of Inhibition Sensitive CLSI (mm)	Zones of Inhibition Intermediate CLSI (mm)	Zones of Inhibition Resistant CLSI (mm)
1	Ciprofloxacin (5 µg)	21 ± 0.1	≥21	16–20	≤15
2	Levofloxacin (5 µg)	14 ± 0.3	≥15	13–14	≤12
3	Amikacin (30 µg)	17 ± 0.2	≥18	14–17	≤13
4	Cefoxitin (30 µg)	17 ± 0.1	≥22	-	≤21
5	Penicillin (10 µg)	6 ± 0.5	≥15	13–14	≤12
6	Linezolid (10 µg)	24 ± 0.2	≥21	-	≤20
7	Co-trimoxazole (25 µg)	6 ± 0.4	≥16	11–15	≤10

Intermediate zones of inhibition can fall between intermediate sensitive or intermediate resistant; standard deviation from mean indicated by ± sign; Clinical Laboratory Standard Institute (CLSI).

**Table 4:** *Enterococcus faecalis* AST (antibiotic susceptibility tests) results in comparison with the standard guidelines of CLSI (Clinical Laboratory Standard Institute 2020).

SR#	Antibiotics Used for *Enterococcus faecalis*	Zones of Inhibition for *Enterococcus faecalis* (mm)	Zones of Inhibition Sensitive CLSI (mm)	Zones of Inhibition Intermediate CLSI (mm)	Zones of Inhibition Resistant CLSI (mm)
1	Ciprofloxacin (5 µg)	13 ± 0.2	≥21	16–20	≤15
2	Levofloxacin (5 µg)	10 ± 0.4	≥17	14–16	≤13
3	Penicillin (10 µg)	8 ± 0.5	≥15	13–14	≤12
4	Amoxicillin (10 µg)	6 ± 0.1	≥18	14–17	≤13
5	Linezolid (30µg)	19 ± 0.3	≥23	21–22	≤20
6	Imipenem (10 µg)	14 ± 0.2	≥21	19–21	≤18
7	Vancomycin (30 µg)	10 ± 0.1	≥17	15–16	≤14

**Table 5:** *Acinetobacter baumannii* AST (antibiotic susceptibility tests) results in comparison with the standard guidelines of CLSI (Clinical Laboratory Standard Institute 2020).

SR#	Antibiotics Used for *Acinetobacter* *baumannii*	Zones of Inhibition for *Acinetobacter* *baumannii* (mm)	Zones of Inhibition Sensitive CLSI (mm)	Zones of Inhibition Intermediate CLSI (mm)	Zones of Inhibition Resistant CLSI (mm)
1	Ciprofloxacin (5 µg)	6 ± 0.3	≥26	22–25	≤21
2	Levofloxacin (5 µg)	9 ± 0.1	≥21	17–20	≤16
3	Amikacin (30 µg)	18 ± 0.3	≥18	14–17	≤13
4	Ceftriaxone (30 µg)	6 ± 0.2	≥23	20–22	≤19
5	Ceftazidime (30µg)	6 ± 0.4	≥21	18–20	≤17
6	Imipenem (10 µg)	6 ± 0.3	≥23	20–22	≤19
7	Co-trimoxazole (25 µg)	19 ± 0.1	≥16	11–15	≤10

**Table 6:** *Klebsiella pneumoniae* AST (antibiotic susceptibility tests) results in comparison with the standard guidelines of CLSI (Clinical Laboratory Standard Institute 2020).

SR#	Antibiotics Used for *Klebsiella* *pneumoniae*	Zones of Inhibition for *Klebsiella* *pneumoniae* (mm)	Zones of Inhibition Sensitive CLSI (mm)	Zones of Inhibition Intermediate CLSI (mm)	Zones of Inhibition Resistant CLSI (mm)
1	Ciprofloxacin (5 µg)	6 ± 0.4	≥26	22–25	≤21
2	Levofloxacin (5 µg)	6 ± 0.3	≥21	17–20	≤16
3	Amikacin (30 µg)	6 ± 0.2	≥18	14–17	≤13
4	Ceftriaxone (30 µg)	6 ± 0.3	≥23	20–22	≤19
5	Ceftazidime (30 µg)	8 ± 0.1	≥21	18–20	≤17
6	Imipenem (10 µg)	7 ± 0.1	≥23	20–22	≤19
7	Co-trimoxazole (25 µg)	6 ± 0.2	≥16	11–15	≤10

**Table 7:** *Pseudomonas aeruginosa* AST (antibiotic susceptibility tests) results in comparison with the standard guidelines.

SR#	Antibiotics Used for *Pseudomonas* *Aeruginosa*	Zones of Inhibition for *Pseudomonas* *aeruginosa* (mm)	Zones of Inhibition Sensitive CSLI (mm)	Zones of Inhibition Intermediate CSLI (mm)	Zones of Inhibition Resistant CSLI (mm)
1	Ciprofloxacin (5 µg)	6 ± 0.4	≥26	22–25	≤21
2	Levofloxacin (5 µg)	6 ± 0.3	≥21	17–20	≤16
3	Amikacin (30 µg)	6 ± 0.2	≥18	14–17	≤13
4	Ceftriaxone (30 µg)	6 ± 0.3	≥23	20–22	≤19
5	Ceftazidime (30µg)	6 ± 0.1	≥21	18–20	≤17
6	Imipenem (10 µg)	6 ± 0.1	≥23	20–22	≤19
7	Co-trimoxazole (25 µg)	6 ± 0.2	≥16	11–15	≤10

**Table 8:** *Achyranthes aspera* seed AST (antibiotic susceptibility tests) results against selected bacterial strains.

*Achyranthes aspera* Seed Extracts	Zone of Inhibition *MRSA* (mm)	Zone of Inhibition *E. faecalis* (mm)	Zone of Inhibition *A. baumannii* (mm)	Zone of Inhibition *K. pneumoniae* (mm)	Zone of Inhibition *P. aeruginosa* (mm)
Methanolic extract	6 ± 0	6 ± 0	6 ± 0	6 ± 0	6 ± 0
Petroleum ether extract	6 ± 0	6 ± 0	6 ± 0	6 ± 0	6 ± 0
Chloroform extract	6 ± 0	6 ± 0	6 ± 0	6 ± 0	6 ± 0
Ethyl acetate extract	6 ± 0	6 ± 0	6 ± 0	6 ± 0	6 ± 0
Aqueous extract	6 ± 0	6 ± 0	6 ± 0	6 ± 0	6 ± 0

Standard deviation from mean indicated by ± sign.

**Table 9:** *Achyranthes aspera* leaves AST (antibiotic susceptibility tests) results against selected bacterial strains.

*Achyranthes aspera* Leaf Extracts	Zone of Inhibition *MRSA* (mm)	Zone of Inhibition *E. faecalis* (mm)	Zone of Inhibition *A. baumannii* (mm)	Zone of Inhibition *K. pneumoniae* (mm)	Zone of Inhibition *P. aeruginosa* (mm)
Methanolic extract	6 ± 0	6 ± 0	6 ± 0	6 ± 0	6 ± 0
Petroleum ether extract	6 ± 0	6 ± 0	6 ± 0	6 ± 0	6 ± 0
Chloroform extract	6 ± 0	6 ± 0	6 ± 0	6 ± 0	6 ± 0
Ethyl acetate extract	6 ± 0	6 ± 0	6 ± 0	6 ± 0	6 ± 0
Aqueous extract	6 ± 0	6 ± 0	6 ± 0	6 ± 0	6 ± 0

Standard deviation from mean indicated by ± sign.

## Data Availability

Not applicable.
